# A cost-utility analysis of cervical cancer screening and human papillomavirus vaccination in the Philippines

**DOI:** 10.1186/s12889-015-2046-1

**Published:** 2015-07-30

**Authors:** Anna Melissa Guerrero, Anne Julienne Genuino, Melanie Santillan, Naiyana Praditsitthikorn, Varit Chantarastapornchit, Yot Teerawattananon, Marissa Alejandria, Jean Anne Toral

**Affiliations:** Department of Health Philippines, Pharmaceutical Division, 3/F Building 15, San Lazaro Compound, Rizal Avenue, Sta. Cruz, 1003 Manila, Philippines; Product Team for Special Benefits, Philippine Health Insurance Corporation, Pasig, 1603 Philippines; Health Intervention and Technology Assessment Program, Ministry of Public Health, Nonthaburi, 11000 Thailand; Institution of Clinical Epidemiology, University of the Philippines, Manila, 1000 Philippines; Obstetrics and Gynecology, Philippine General Hospital, Manila, 1000 Philippines

**Keywords:** Human papilloma virus, Vaccination, Cervical neoplasms, Immunization, Vaccines, Cervical cancer screening, Low-middle country, Visual inspection, Pap smear, Cost-utility analysis, Economic evaluation

## Abstract

**Background:**

Cervical cancer is the second leading cause of cancer cases and deaths among Filipino women because of inadequate access to screening and treatment services.  This study aims to evaluate the health and economic benefits of HPV vaccination and its combination with different screening strategies to find the most optimal preventive strategy in the Philippines.

**Methods:**

A cost-utility analysis was conducted using an existing semi-Markov model to evaluate different screening (i.e., Pap smear, visual inspection with acetic acid) and vaccination strategies against HPV infection implemented alone or as part of a combination strategy at different coverage scenarios. The model was run using country-specific epidemiologic, cost and clinical parameters from a health system perspective. Sensitivity analysis was performed for vaccine efficacy, duration of protection and costs of vaccination, screening and treatment.

**Results:**

Across all coverage scenarios, VIA has been shown to be a dominant and cost-saving screening strategy with incremental cost-effectiveness ratio (ICER) ranging from dominant to Php 61,059 (1443 USD) per QALY gained. VIA can reduce cervical cancer cases and deaths by 25 %. Pap smear screening was found to be not cost-effective due to its high cost in the Philippines. Adding HPV vaccination at a cost of 54 USD per vaccinated girl on top of VIA screening was found to be potentially cost-effective using a threshold of 1 GDP per capita (i.e., Php 120,000 or 2835 USD/ QALY) with the most favorable assumption of providing lifelong immunity against high-risk oncogenic HPV types 16/18. The highest incremental QALY gain was achieved with 80 % coverage of the combined strategy of VIA at 35 to 45 years old done every five years following vaccination at 11 years of age with an ICER of Php 33,126 (783 USD). This strategy may result in a two-thirds reduction in cervical cancer burden. HPV vaccination is not cost-effective when vaccine protection lasts for less than 20 years.

**Conclusion:**

High VIA coverage targeting women aged 35–45 years old at five-year intervals is the most efficient and cost-saving strategy in reducing cervical cancer burden in the Philippines. Adding a vaccination program at high coverage among 11-year-old girls is potentially cost-effective in the Philippines assuming a life-long duration of vaccine efficacy.

**Electronic supplementary material:**

The online version of this article (doi:10.1186/s12889-015-2046-1) contains supplementary material, which is available to authorized users.

## Background

Cervical cancer is the second most common female cancer in the Philippines with about 6670 diagnosed cases in 2010 and an annual age-standardized incidence rate of 11.7 per 100,000 women [1]. Between 1980 and 2010, the overall 5-year survival rate has not improved at 44 % because of late-stage diagnosis as a result of the lack of screening and inadequate treatment services [2–5].

Pap smear was first introduced in the Philippines in the 1990’s for women 35 to 55 years old done once in a lifetime [6]. In 2005, the policy was shifted to the single visit approach using visual inspection with acetic acid (VIA) followed by cryotherapy because this was a more practical approach than Pap smear which had a very low uptake at 7.7 % [7, 8]. The current national recommendation is to target women 25–55 years with VIA done at five to seven year intervals. Colposcopy with Pap smear or biopsy was only recommended as a confirmatory diagnostic test following a positive VIA test.

It is estimated that HPV types 16 and 18 together contribute to 70 % of all invasive cervical cancer cases worldwide [9–11]. This is also observed among Filipino women where both types are also predominant (see Additional file 1: Figure S1) [12, 13]. Prophylactic vaccination against persistent HPV infection offers an alternative preventive strategy against cervical cancer particularly in developing countries which lack a nationally organized cervical screening program. Two vaccines are currently registered by the Philippine FDA, a bivalent vaccine (Cervarix®) and a quadrivalent vaccine (Gardasil®), both protecting against high-risk oncogenic types HPV 16 and 18 which cause majority of cervical cancers as well as other associated vaginal, vulvar, penile, anal and oropharyngeal cancers that are less common in the Philippines [14].

The World Health Organization recommends the introduction of HPV vaccination as part of a comprehensive national cervical control program in settings where cervical cancer is a public health priority and where the feasibility and cost-effectiveness of HPV vaccines have been considered [15]. This study aims to inform Filipino policy makers on the health and economic benefits and the financial requirements of different preventive strategies against cervical cancer. Given that different screening options and HPV vaccination will have considerable resource impact to the local health system, there is a need for evidence to ensure the efficient allocation of funding toward optimal preventive strategies against cervical cancer especially when budgets are constrained.

## Methods

### Study design

The study is a cost-utility analysis adapted from an existing static model applied previously in the Thai setting comparing the cost-effectiveness of different preventive programs against cervical cancer. The model enables an evaluation of different screening and vaccination strategies implemented alone or as part of a combination strategy with varying coverage scenarios [16, 17]. We ran the model using country-specific epidemiologic, cost and clinical parameters via Microsoft Excel 2007 spreadsheet (Microsoft Corp., Redmond, WA, USA).

We used a health system perspective as the primary analysis in this study incorporating the program costs of vaccination, screening and public hospital services to treat cervical cancer in the Philippines. The main health effect of the interventions was measured in terms of healthy life days gained and quality adjusted life years (QALYs). We also reported health outcomes in natural units as the number of cases and deaths due to invasive cervical cancer. A lifetime horizon was used through a Monte-Carlo simulation to cover the expected survival time of the female cohort in this study (i.e., females 11 years old and above) [18–20]. All cost and outcome parameters were discounted at 3.5 % per annum based on current recommendations recently approved by the Philippine Formulary Executive Council [21].

### Interventions and assumptions

#### HPV Vaccination

The vaccination strategy was analyzed as an add-on strategy to the existing screening program based on international recommendations that it should be introduced as part of a comprehensive preventive program and does not replace conventional screening methods particularly in low-resource settings [15]. A school-based strategy was considered in the model because of the large catchment of this approach among targeted pre-adolescent girls.

The study assumed equal protective efficacy against high-risk HPV types for the two existing vaccines and therefore similar efficacy in preventing cervical cancer. We modeled different scenarios where there is limited and lifetime protection using a three-dose regimen of existing vaccines with additional scenarios that booster doses would be needed every 0, 10, 15 and 20 years. The start age of vaccination was modeled at 11, 12 and 13 years old prior to sexual debut. We also explored expanding vaccine coverage to women at 20 and 25 years of age to determine the cost-effectiveness of this strategy while acknowledging that a significant proportion of these women are not likely to be naïve to the vaccine-related HPV types.

#### Cervical cancer screening

In this study, we reviewed the existing policy on Pap smear and modeled different start ages of screening at 20, 25, 30 and 35 years of age until 55, 60 or 65 years at five-year intervals. Single visit approach with VIA plus cryotherapy was also modeled at different start ages of 20, 25, 30 and 35 done every five years until 45, 50 and 55 years of age.

We also considered the option that Pap smear is done as a complementary strategy to VIA starting at the age of 50 until 55, 60 or 65 years of age at five-year intervals. The most cost-effective strategy was chosen as the dominant strategy. The base case scenario was taken at the current low coverage scenario of Pap smear at 8 % for women 35 to 55 years old done at five-year intervals [2, 3].

#### Combination strategies

We compared the performance of different options to implement cervical cancer prevention programs in the Philippines (i.e., three-dose vaccination with or without booster doses, conventional Pap smear alone and VIA alone) (see Additional file 2: Table S1). We also combined different strategies to look for the optimal mix of preventive programs that could be recommended for wide scale adoption.

Since program effectiveness for cervical cancer prevention strategies is a function of coverage, we assumed ‘low’ and ‘high’ coverage scenarios whereby ‘low coverage’ was set at the current 8 % for Pap smear and VIA and 20 % for vaccination assuming that only the poorest quintile of schoolgirls could be covered by a publicly funded vaccination program. ‘High coverage’ was set at 80 % for both screening services and vaccination under the assumption that the government has available funding to scale them up either as individual strategies or as part of a comprehensive cervical cancer program (Table 1).

**Table 1:** Assumptions in coverage scenarios for HPV vaccination and screening as used in the economic modeling

Scenarios	Vaccination	Screening
Scenario I – Worst case	20 % (poorest quintile)	8 %
Scenario II	20 %	80 %
Scenario III	80 %	8 %
Scenario IV – Best case	80 %	80 %

For each coverage scenario, we identified optimal approaches defined as those having the lowest incremental cost-effectiveness ratios (ICERS) calculated as the additional cost of the incremental benefit of one strategy compared to the next less costly strategy. More costly and less effective strategies were considered ‘dominated’ strategies and eliminated from further analysis. All possible single and combination approaches were compared with the base case scenario of Pap Smear at 8 % coverage for women aged 35–55 years old at 5-year intervals.

## Model parameters

**Table 2:** Parameters used in the model and their sampling distribution for the probabilistic sensitivity analysis

Parameters	Mean (SE)	Distribution	Reference
Baseline Parameters			
Discount Rate for both costs and outcomes	3.5 %		DOH, 2013
Age start in the model (years)	11		
PPP conversion factor, (Pesos per 1$)	24.8		World data bank, 2013
Epidemiological Parameters			
Prevalence of HPV infection	0.100 (0.064)	Beta	Myers et al. [22]
Prevalence of CIN1	0.010 (0.010)	Beta	Myers et al. [22]
Age specific (y) incidence of HPV infection			
11	0.019 (0.007)		
15	0.100 (0.038)	Beta	Myers et al. [22]
16	0.100 (0.038)	Beta	
17	0.120 (0.046)	Beta	
18	0.150 (0.057)	Beta	
19	0.170 (0.065)	Beta	
20	0.150 (0.057)	Beta	
21	0.120 (0.046)	Beta	
22	0.100 (0.038)	Beta	
23	0.100 (0.038)	Beta	
24	0.050 (0.019)	Beta	
30	0.010 (0.004)	Beta	
50+	0.005 (0.002)	Beta	
Yearly Transitional Probability			
HPV infection to CIN1	0.072 (0.015)	Beta	Myers et al. [22]
CIN1 to CIN2/3 (age [y])			
15	0.017 (0.010)	Beta	Myers et al. [22]
35	0.069 (0.013)	Beta	
CIN 2/3 to invasive CA	0.050 (0.008)	Beta	
Stage I to Stage II	0.438 (0.351)	Beta	Myers et al. [22]
Stage II to Stage III	0.536 (0.351)	Beta	
Stage III to Stage IV	0.684 (0.140)	Beta	
Regression			
Age-specific (y) probability of regression: HPV infection to healthy			Myers et al. [22]
15	0.552 (0.084)	Beta	
25	0.370 (0.033)	Beta	
30	0.103 (0.018)	Beta	
Age-specific (y): CIN1 to HPV infection or healthy			Myers et al. [22]
15	0.161 (0.024)	Beta	
35	0.082 (0.021)	Beta	
CIN 2/3 to CIN1 or healthy	0.069 (0.013)	Beta	Myers et al. [22]
Proportion of CIN1 reverting to healthy	0.900 (0.128)	Beta	
Proportion of CIN2/3 reverting to healthy	0.500 (0.128)	Beta	
Proportion of having symptoms			Myers et al. [22]
Stage I	0.150 (0.150)	Beta	
Stage II	0.225 (0.225)	Beta	
Stage III	0.600 (0.600)	Beta	
Stage IV	0.900 (0.900)	Beta	
Weibull survival by CA stage and patient age (y)			
Stage I			
constant	−8.749 (1.259)	Log-Normal	Praditsitthikorn et al. [17]
Age coefficient	0.041 (0.020)	Log-Normal	
Gamma	0.589 (1.139)	Log-Normal	
Stage II			
constant	−7.066 (0.934)	Log-Normal	
Age coefficient	−0.014 (0.011)	Log-Normal	
Gamma	0.919 (1.120)	Log-Normal	
Stage III			
constant	−6.778 (0.891)	Log-Normal	
Age coefficient	0.023 (0.011)	Log-Normal	
Gamma	0.675 (1.098)	Log-Normal	
Stage IV			
constant	−3.863 (1.217)	Log-Normal	
Age coefficient	−0.055 (0.022)	Log-Normal	
Gamma	1.004 (1.226)	Log-Normal	
Program Effectiveness Parameters			
* Pap Smear*			
Sensitivity for pre-invasive	0.552 (0.070)	Beta	Sritipsukho, [27]
Specificity	0.915 (0.013)	Beta	
* VIA*			
Sensitivity for pre-invasive	0.716 (0.025)	Beta	Sritipsukho, [27]
Specificity	0.793 (0.011)	Beta	
* HPV Vaccine*			
Relative risk of persistence HPV infection, 1-year	0.26 (0.064)	Beta	Rambout et al. [25]
Programme Acceptability			
Pap Smear	0.08		University of the Philippines-Department of Health Cervical Cancer Screening Study Group, 2001 [7]
Proportion of Patients with CIN 2/3			
Receiving cryosurgery	1.000 (1.000)	Beta	Goldie et al. [44]
Receiving cold knife conisation	0.125 (0.125)	Beta	Goldie et al. [44]
Receiving simple hysterectomy	0.125 (0.125)	Beta	Goldie et al. [44]
Incidence of OP visit for treating minor complications from cryosurgery	0.05 (0.05)	Beta	Goldie et al. [44]
Incidence of IP visit for treating major complications from cryosurgery	0.01 (0.01)	Beta	Goldie et al. [44]
Annual rate of OP visits			Praditsitthikorn et al. [17]
Initial Stage	25.48 (1.41)	Gamma	
Remission Stage	7.14 (0.59)	Gamma	
Persistence Stage	38.53 (7.77)	Gamma	
Recurrence Stage	13.37 (2.02)	Gamma	
Annual rate of IP visits			Praditsitthikorn et al. [17]
Initial Stage	0.77 (0.10)	Gamma	
Remission Stage	0.15(0.04)	Gamma	
Persistence Stage	0.87 (0.43)	Gamma	
Recurrence Stage	1.64 (0.31)	Gamma	
Costing Parameters (in Php)			
Direct Medical Costs of Screening (per visit)			
Pap smear	965 (965)	Gamma	Primary data collected by the authors
VIA	500 (500)	Gamma	Primary data collected by the authors
Cost of follow up for Pap screening	500 (500)	Gamma	Primary data collected by the authors
Cost of HPV vaccination (three doses)	2,736 (2,376)	Gamma	Price Offer to the government
Cost of HPV booster doses	800 (800)	Gamma	Price Offer to the government
Cost of Vaccine delivery and administration (per dose)	112 (112)	Gamma	DOH DPCB, 2013
Unit cost of colcoscopy/ biopsy	1,120 (1,120)	Gamma	PHIC, 2013
Unit costs			
Cryotherapy	1,500 (1,500)	Gamma	Primary data collected by the authors
Loop Electrosurgical Extraction Procedure (LEEP)	12,644.54 (12,644.54)	Gamma	PHIC, 2013
Cold knife conisation	8100.36 (8100.36)	Gamma	PHIC, 2013
Simple hysterectomy	41,362.67 (41,362.67)	Gamma	PHIC, 2013
Cost of hospitalization day (Php per day)	500 (500)	Gamma	Health facilities
Hospitalization days			
Cold knife conisation	1	Gamma	Expert opinion
Simple hysterectomy	5	Gamma	Expert opinion/ Primary data collected by the authors
Medical cost of follow – up			
Cryosurgery	1,000 (255.10)	Gamma	Primary data collected by the authors
LEEP/ Cold knife conisation/ Simple hysterectomy	750 (127.55)	Gamma	Primary data collected by the authors
Unit Cost			
Cervical CA staging	4,485 (765.31)	Gamma	Primary data collected by the authors
Treating complications from cryosurgery (minor)	510.08 (510.08)	Gamma	Primary data collected by the authors
Treating complications from cryosurgery (major)	512.48 (512.48)	Gamma	Primary data collected by the authors
Annual Costs for treatment of invasive cervical CA			
Initial Stage			
-Stage I	77,873.00 (39,073.469)		PHIC 2013
-Stage II	77,873.00		PHIC 2013
-Stage III	106,390.05		PHIC 2013
-Stage IV	106,390.05		PHIC 2013
Remission Stage			
-Stage I	16,523		PHIC 2013
-Stage II	16,115		PHIC 2013
-Stage III	20,618		PHIC 2013
-Stage IV	27,310		PHIC 2013
Persistence Stage			
-Stage I	112,093		PHIC 2013
-Stage II	93,256		PHIC 2013
-Stage III	118,350		PHIC 2013
-Stage IV	117,801		PHIC 2013
Recurrence			
-Stage I	65,818		PHIC 2013
-Stage II	63,747		PHIC 2013
-Stage III	83,512		PHIC 2013
-Stage IV	111,233		PHIC 2013
Utility Parameters			
Healthy Stage or CIN1-3 without complication	1.00 (1.00)	Beta	Praditsitthikorn et al. [17]
Initial Stage			
-Stage I	0.74 (0.01)	Beta	
-Stage II	0.76 (0.01)	Beta	
-Stage III	0.72 (0.02)	Beta	
-Stage IV	0.63 (0.03)	Beta	
Remission Stage			
-Stage I	0.79 (0.01)	Beta	
-Stage II	0.79 (0.01)	Beta	
-Stage III	0.81 (0.01)	Beta	
-Stage IV	0.85 (0.05)	Beta	
Persistence Stage			
-Stage I	0.80 (0.20)	Beta	
-Stage II	0.80 (0.04)	Beta	
-Stage III	0.65 (0.05)	Beta	
-Stage IV	0.45 (0.05)	Beta	
Recurrence			
-Stage I	0.80 (0.03)	Beta	
-Stage II	0.68 (0.02)	Beta	
-Stage III	0.66 (0.04)	Beta	
-Stage IV	0.81 (0.08)	Beta	

DOH - Department of Health; DPCB - Disease Prevention and Control Bureau; PHIC - Philippine Health Insurance Corporation

### Baseline parameters

#### Transitional probabilities

**Fig. 1:**
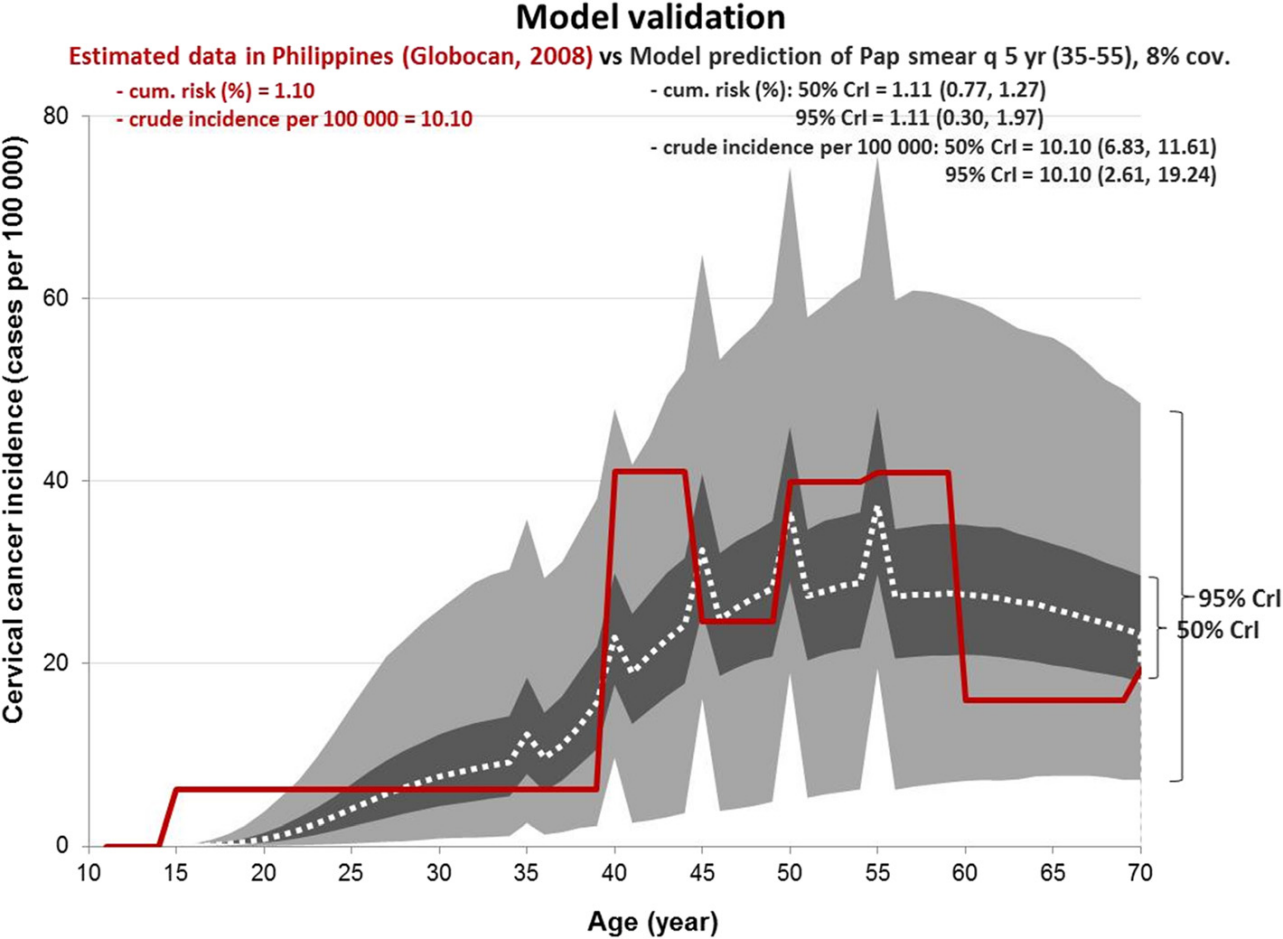
Model validation

The semi-Markov model shows the natural history of cervical cancer including the transitional probabilities of progressing to different stages of the disease. In the absence of local data in the Philippines, transition rates on the natural history and progression of cervical cancer due to HPV-related infections and age-specific HPV incidence rates were adopted from the study of Myers et al. (Table 2) [22]. The model was validated at 8 % screening coverage. We calibrated the model by adjusting the HPV infection rate to closely fit the observed data on age-specific annual incidence of cervical cancer in the Philippines reported in the 2008 WHO GLOBOCAN database [1]. Cancer statistics in this database are derived from the recorded population-based estimates from the Manila and Rizal Cancer registries, which are regarded as among the high-quality cancer registries by the WHO International Agency for Research on Cancer (IARC) [4]. Figure 1 shows the reliability of the model to predict results based on local cancer incidence input parameters at 95 % credibility interval.

Mortality data for the Filipino general population were obtained from the WHO life tables for the year 2011 [23]. Age- and stage–specific survival rates were calculated from a retrospective study by Redaniel et al. among 1,580 Filipino patients randomly selected from the national population-based cancer registries during the period 1993–2002 followed up with respect to vital status [24]. Parametric analysis of the Weibull survival distribution among Filipino patients with remitting, persistent and recurrent disease at Stage I, II, II and IV disease was done using methods already described previously [17].

### Clinical effectiveness

Data on the clinical efficacy of existing HPV vaccines on cervical cancer was adopted from the meta-analysis of six randomized controlled trials done by Rambout et al. which reported a vaccine efficacy of 74 % (95 % CI: 59-84 %) in reducing persistent HPV infection at 12 months [25]. We derived estimates on the number of eligible girls for vaccination for the year 2015 from the Philippine National Statistical Coordination Board (NSCB) population projection data [26].

We obtained data on the accuracy and test performance of VIA and Pap smear from a systematic review done by Sritipsukho et al. based on a pooled analysis of studies mostly conducted in developing country settings. In this study, VIA was found to have low accuracy at the pre-invasive stage with sensitivity and specificity of 71.6 % (SE = 2.5 %) and 79.3 % (SE = 1.1 %). Further, the technique is known to have low accuracy in detecting lesions among postmenopausal women in whom lesions may not be visible on speculum inspection. A lower sensitivity of 55 % (SE = 7 %) was noted for Pap smear although specificity was high at 91.5 % (SE = 1.3 %) based on a meta-analysis of 15 studies [27].

Currently, no data could be obtained for the coverage status of VIA but it was assumed at the same level as that of Pap smear as training of providers on VIA has not been scaled up on a national level.

### Utility estimates

Because of the absence of utility estimates on the quality of life of Filipino cervical cancer patients, we derived data from a study involving a cohort of 1,035 Thai women with invasive cervical cancer seen in regional cancer centers and university hospitals in Thailand. In this study, it was found that the lowest utility value were from women with persistent Stage IV disease while the highest utility scores were obtained from patients in the remission state for all stages of cancer. The utility values were measured using the VAS elicitation method, which have been, described previously [28–30].

### Cost data

Cost parameters included in this model consist of direct medical costs due to vaccination, screening and treatment of pre-cancerous and cancerous stages in the public health care system. All costs were reported in Philippine peso and converted to 2013 values using the Philippine Consumer Price Index for health services and adjusted for inflation [31]. Costs were also converted to US dollars using the mean exchange rate between the US dollar and the Philippine peso in 2013 (1 USD = Php 42.32) [32].

Costs associated with implementing a HPV vaccination program were calculated using a school-based strategy targeting 11-year-old girls with 80 % coverage in the best case scenario (more than 800,000 annually) receiving the full three doses. We also considered an alternative scenario where the uptake of the vaccine is low at 20 % coverage. The unit cost of the vaccine was calculated at Php 800 (19 USD) per dose which is the current list price offered by a vaccine manufacturer to the Department of Health (DOH) [33]. Other cost components for HPV vaccination were derived from historical data on existing national immunization programs: 8 % storage, freight and distribution; 1 % other program costs (i.e., other supplies, surveillance, community engagement and training), and; 5 % wastage cost [34]. Costs due to health complications following vaccination were not included since we assumed that side effects of the vaccine are minimal.

Screening costs include the cost of Pap smear and VIA procedures derived from one major referral tertiary public hospital validated through nominal group technique and a structured costing questionnaire given to a group of gyne-oncologic experts in the Philippines. We assumed two clinic visits for Pap Smear while VIA requires only one clinic visit. Other screening costs include the cost of supplies and an additional reading fee for the cytological analysis of Pap smear.

The costs of treatment of CIN 1/2/3 cervical disease were derived from the average amount of claims for cervical procedures and the Relative Value Scale (RVS) of the Philippine Health Insurance Corporation (PHIC) for 2011 and 2012. These include cryotherapy, loop electrosurgical extraction procedure (LEEP), cold knife conization and simple hysterectomy. For the treatment of invasive cervical cancer, we used the 2013 standard case rates of Philhealth calculated through activity-based-costing of services using locally accepted clinical guidelines [35, 36]. Drug costs were based on the drug price reference index of the DOH reflecting the median price of acquisition of drugs in public hospitals adjusted upwards by 10 % to incorporate pharmacy administration services [37]. Some data on health resource use were adopted from the Thai setting for lack of local data. These include patterns of ambulatory care, hospital visits as well as type of treatment received by patients with pre-malignant and malignant disease derived from 12 cancer centers and university hospitals in Thailand [38].

### Uncertainty analyses

In this study, we performed probabilistic sensitivity analysis (PSA) to capture the variability surrounding the input parameters used in the model, which include estimates on transitional probabilities, survival rates, costs and utility values. Table 2 shows the choice of distribution for each input parameter used in the model with the justification of the using the distribution already described elsewhere [39]. Using probabilistic methods, we captured uncertainty in the results with 10,000 Monte-Carlo simulations across the respective probability distributions of the different parameters [39–41].

We also ran a threshold analysis under a high screening coverage scenario to determine a cost-effective price for the HPV vaccine given the need for additional booster doses (i.e., every 0, 10, 15 and 20 years). A ceiling threshold of Php 120,000 or 2,835 USD (i.e., 1 x GDP per capita) was used to reflect the maximum willingness to pay (WTP) of decision-makers for an additional QALY based on current Philippine guidelines. We also assumed a ceiling threshold of zero to reflect the probability that local decision-makers are not willing to pay additional costs for a QALY gained because of existing budget constraints and would want to prioritize interventions that are potentially cost-saving [41].

To identify which model parameters have the most influence in our model results, we conducted one-way sensitivity analyses for vaccine efficacy, duration of vaccine protection, test performance of VIA and Pap smear, cost of vaccine, cost of Pap smear, cost of VIA, cost of treatment of pre-cancerous and cancerous lesions, discount rate and utility scores of patients as these parameters were deemed to have the greatest uncertainty in the model. We calculated the Incremental Cost-Effectiveness Ratios (ICERs) and percent change from the reference ICER comparing 80 % coverage of VIA done at 35–55 years old and 80 % coverage of the combination of VIA at 25–55 years old and Pap smear at 60–65 years of age under the low vaccine coverage (20 %) scenario. The values reported represent the 2.5 percentile and 97.5 percentile credibility intervals [39–41].

## Results

### Optimal policy options for cervical cancer prevention

**Fig. 2:**
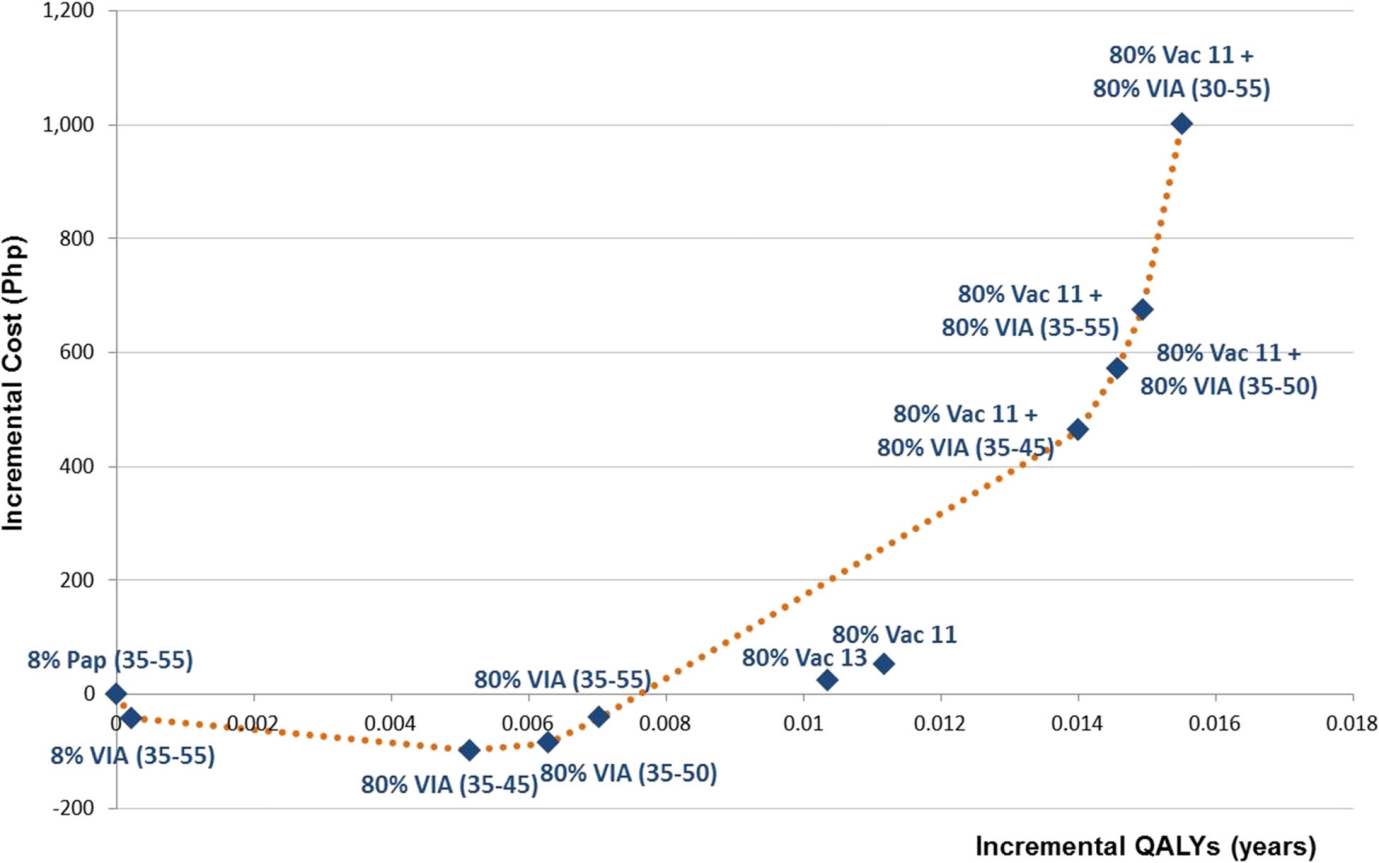
Efficiency frontier curve of optimal cervical cancer prevention strategies at varying coverage scenarios

**Fig. 3:**
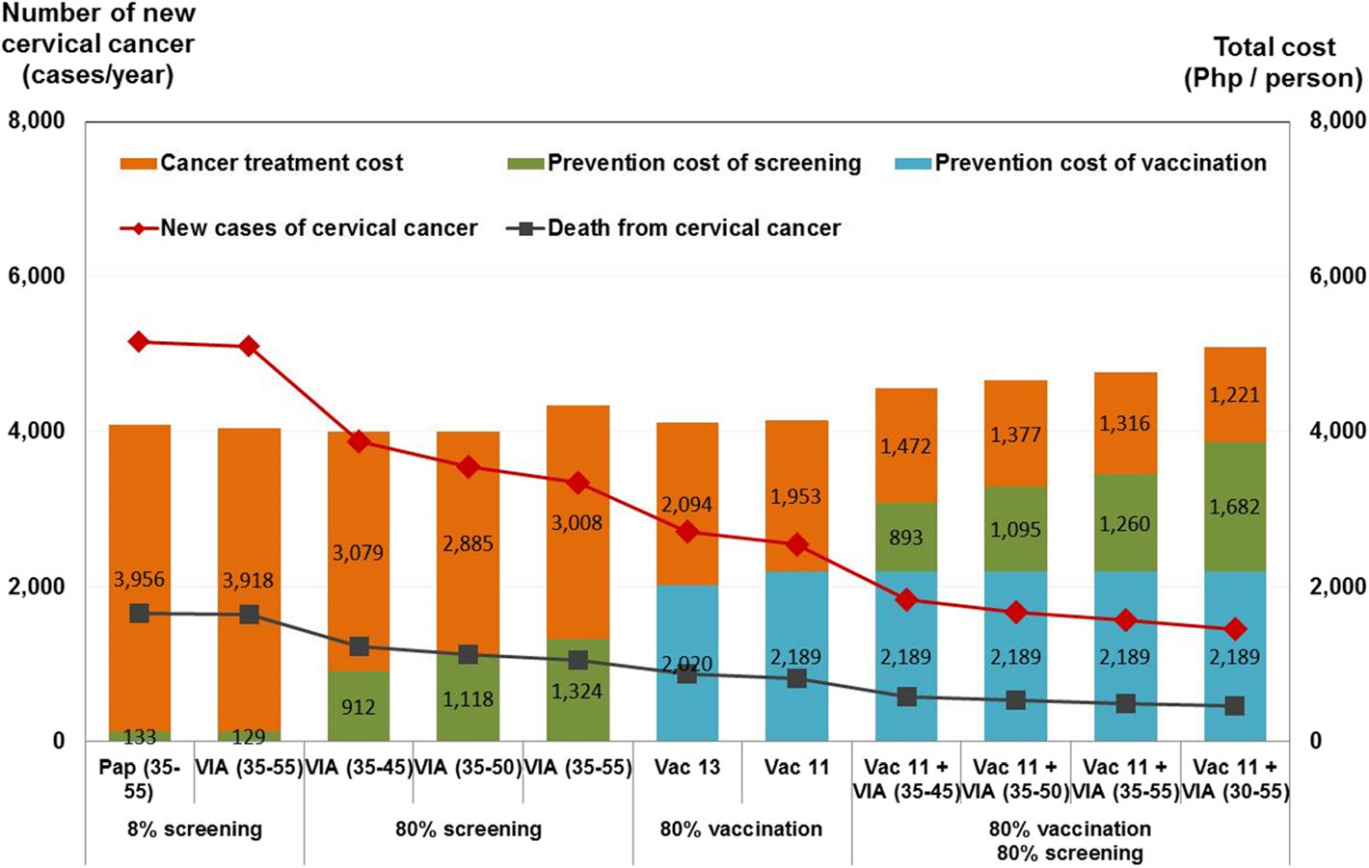
Costs and health outcomes of optimal strategies for the prevention of cervical cancer

Figure 2 shows the efficiency frontier curve of eight optimal strategies across different coverage scenarios defined as those that are more effective and less costly compared to the next best competing strategy. More detailed results of the optimal strategies on total costs, total life years gained, total QALYs, total cervical cancer cases averted, number of deaths prevented and ICERs are shown in Fig. 3 and discussed below.

Across all coverage scenarios, VIA has been shown to be a cost-saving strategy with ICERs ranging from dominant to Php 61,059 (1,443 USD) per QALY gained. The negative cost per QALY ratios indicate that compared to the base case scenario at low Pap smear coverage, shifting to VIA will be cost-saving to the government and accrue higher benefits as coverage is expanded.

The most efficient screening option will be performing VIA at 35 to 45 years old done every five years with this approach achieving greater incremental QALYs at lower costs. The cost of the screening program becomes more expensive as the age range of women targeted for screening is widened and the frequency of testing is increased from three to six times per lifetime.

Strategies involving Pap smear done alone or in combination with other strategies were found to be not cost-effective in the Philippines across all coverage scenarios and were therefore dominated in all analyses.

### Cost-effectiveness of combination strategies

Considering lifelong protective immunity, adding HPV vaccination to VIA done three times per lifetime at 35 to 45 years old is potentially cost-effective when 80 % coverage is achieved for the combination strategy (Fig. 2). This strategy results in the highest clinical benefit with more than 60 % reduction in the number of cervical cancer cases and deaths although these benefits are likely to be seen decades after the target cohort of girls receive HPV vaccination (Fig. 3). However, implementing HPV vaccination at low coverage (i.e., 20 %) was dominated in all scenarios because of the substantially lower clinical benefits.

Widening the age of VIA screening above 45 years old will have smaller incremental clinical benefits while the costs disproportionately escalate with increased screening frequency leading to step-wise ICERs that exceed the threshold (Fig. 2).

Vaccination at 11 and 13 years of age were found to be cost-effective under favourable conditions of lifelong immunity. Vaccination at 20 and 25 years of age were shown to have cost-effectiveness ratios that exceeded the threshold and are therefore not cost-effective. Our analysis also shows that HPV vaccination is not cost-effective when vaccine protection lasts for less than 20 years (See Additional file 3: Figure S2).

We also performed threshold analysis to calculate the ceiling price per dose of the vaccine to incorporate the uncertainty in the longevity of vaccine efficacy. With a ceiling ratio set at the current threshold of Php 120, 000 or 2,835 USD, the ceiling price per dose was calculated at Php 1,050 (25 USD) assuming lifetime protection against HPV 16/18 infection. With waning vaccine efficacy, the government is less willing to pay a higher ceiling price per dose because of the lower net benefit of HPV vaccination weighed against the additional cost and logistical challenges associated with further doses of the vaccine. For example, assuming vaccine-induced protection of up to 10 years and the need for booster doses, the ceiling price per dose of the vaccine decreases up to Php 386 (9 USD) with the need for 3 booster doses.

In the scenario where the ceiling threshold is equal to zero, the WTP of the government is Php 702 (17 USD) with the assumption that the vaccine confers lifetime protection. With only 10 years of assured immunity against HPV 16/18 infection, the ceiling price per dose of the vaccine decreases up to Php 258 (6 USD) if 3 additional booster doses are required (See Additional file 4: Table S2).

### Sensitivity analyses

**Fig. 4:**
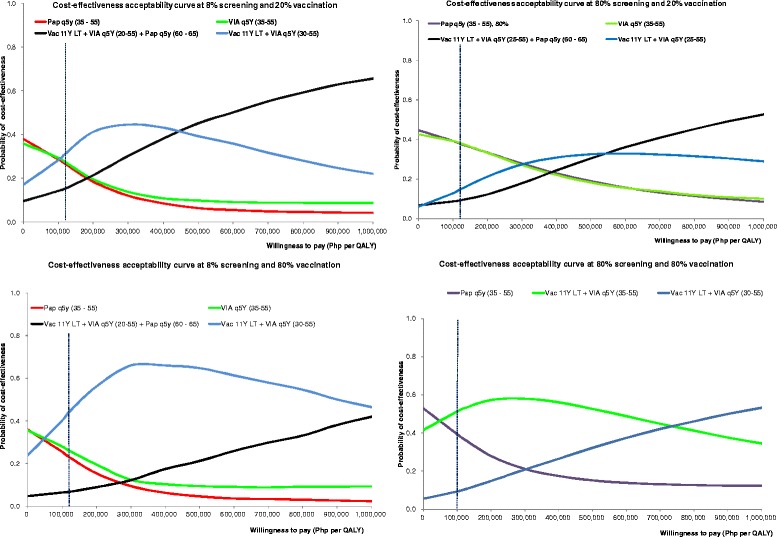
Probabilistic sensitivity analysis of the optimal policy options at different scenarios

Figure 4 show the results of our probabilistic sensitivity analyses where we plotted hypothetical estimates of the ceiling ratios against the probability of new strategies to be cost-effective versus the base case.

As can be seen in Figure 4.1 illustrating the pessimistic scenario of low vaccination and screening coverage, the best policy option is a combination of vaccination at 11 years of age followed by VIA at 30 to 55 years old if Philippine decision makers are no more than willing to pay above the ceiling threshold with the probability of this approach being cost-effective at 31 %. In Fig. 4.2 which illustrates the situation where decision-makers are only prepared to cover the poorest quintile of adolescent girls, the more efficient strategies are to expand screening coverage either through VIA or pap smear at 35 to 55 years old with the probability of each these approaches being more cost-effective at 38 %.

We also explored scenarios where the government is prepared to cover all 11-year-old adolescent schoolgirls at 80 % vaccination coverage. In the low screening coverage scenarios (Figures 4.3), the most cost-effective option is a mixed strategy of vaccination at 11 years old and VIA at 30–55 years old done at five-year intervals. The probability of this approach to be cost-effective is 44 %. Under the optimistic scenario of high vaccine and screening coverage at 80 % (Figure 4.4), the most efficient strategy is VIA screening at 35–50 years old with the probability of being cost-effective at 59 %. If the WTP threshold is higher at Php 280,000 (6616 USD) then the combination of VIA at 30–55 and vaccination at 11 years old becomes the most effective strategy.

The results of the additional one-way sensitivity analysis are shown in Additional file 5: Figure S3. The most influential parameters were the discount rate, cost of treatment, cost of Pap smear, cost of vaccine, duration of vaccine protection and the coverage of screening.

## Discussion

This study strengthens the evidence on the current policy to scale up the coverage of VIA screening in the Philippines as a more efficient strategy compared to conventional Pap smear, which has been impractical to implement in the country. In our model, we projected that shifting from the current low coverage of Pap smear to VIA at high coverage will result in a lower healthcare cost and a higher health benefit. The higher the coverage, the greater the cost saving because VIA is a cost-saving option.

VIA screening at three times per lifetime targeting women 35 to 45 years old done at five-year intervals has the potential to reduce cervical cancer cases and deaths by at least 25 %. Increasing the frequency of VIA to four to six times per lifetime would provide a relative increase in QALY and a decrease in cervical cancer morbidity although the incremental benefits are much smaller leading to stepwise ICERs that exceed the threshold. Filipino decision-makers will have to consider other aspects of VIA as a screening tool apart from program-related costs in broadening the target ages for screening. These include test performance (i.e., low accuracy among women above 45 years old), cultural acceptability and the required training and infrastructure to implement a more inclusive VIA screening policy [42, 43].

Our study is consistent with the previous analysis of Goldie et al. which recommended VIA for women between 35 and 45 years of age as an alternative screening option in developing countries with the potential to reduce cervical cancer incidence by 50 % [44]. The results of our analysis however, contrast with the current national recommendation to target women with VIA screening at 25 to 55 years old [8]. In our analysis, this strategy was dominated because it is much more costly with minimal health benefits.

In this study, we also report that introducing HPV vaccination on top of VIA screening may represent good value for money in the Philippines under favorable assumptions of lifelong protective immunity. However, the cancer benefits of HPV vaccines targeting young girls will not be demonstrated until after decades of implementing HPV vaccination because of the slow natural progression of persistent HPV infection to invasive cervical cancer (ICC) [45–47]. Achieving low coverage of vaccination is unlikely to be cost-effective because of the minimal reduction in the burden of cervical cancer.

The conclusions drawn in this study on VIA screening implemented at high coverage are comparable with the results in Thailand which led to the decision of the Thai policy-makers to prioritize screening over HPV vaccination. In both settings, VIA screening was found to be both a cost-saving and a cost-effective strategy. However, our analysis also differs on several aspects despite the adaptation of the same economic model to the local setting.

First, Pap smear was shown to be an inefficient strategy across all coverage scenarios in the Philippines. This contrasts with the current national cervical prevention strategy in Thailand targeting women aged 50–60 years with Pap smear [16]. The difference in findings may be attributed to the significantly higher costs of Pap smear in the country (i.e., Php 965 or 23 USD) which require about two to three specialist visits in tertiary hospitals in contrast with the organization of screening services in Thailand where Pap smear is more widely available in primary care clinics [16, 17]. Our analysis might be changed if the cost of Pap smear is reduced comparable to the cost in Thailand (i.e., Php115 or 3 USD) and other developing countries.

Second, HPV vaccination was found to be potentially cost-effective on top of VIA screening under the best-case scenario of high vaccine coverage and lifetime protective immunity with an ICER of Php 33,126 (783 USD) per QALY. The previous study in Thailand did not show that vaccination was cost-effective in all possible scenarios [17]. The discrepancy in findings are driven largely by differences in vaccine price and country-specific cost data for treatment services which have been shown in our analysis to be key influential parameters in the model. Our study employed a relatively low HPV vaccine price compared to the analysis conducted in Thailand which used a higher price of the vaccines when they were first introduced in the market. In contrast, we used a significantly lower price offered by vaccine manufacturers to the DOH. In the Philippines, the current market prices of the bivalent and quadrivalent vaccines are comparable with the values used in the original Thai analysis. Therefore, at the prevailing market prices in the country, HPV vaccines would not also give good value for money given the ceiling threshold of 2,835 USD per QALY. Treatment costs for all stages of cervical cancer were also found to be significantly higher in the Philippines making HPV vaccination more attractive because of the higher projected total costs of treatment averted as compared with the Thai setting.

Changing the assumptions on the duration of vaccine efficacy, however, will not also make vaccination a cost-effective strategy if protective immunity lasts for less than 20 years requiring the added expense of more booster doses. At best, current HPV vaccines have shown no waning efficacy for three doses with the longest published trial reporting follow-up of up to 8.4 years for the bivalent vaccine [48]. The evidence using a two-dose schedule, while more attractive for practical and logistical reasons, has even more uncertainty as evidence is based on immunogenicity data with even more limited follow up of up to four years [49].

The government will therefore need to establish post-immunization surveillance to monitor the long-term performance of existing vaccines and prepare a risk management strategy in the event that further booster doses are warranted because of waning efficacy.

Apart from the cost-effectiveness analysis, decision makers will also have to consider the feasibility and sustainability of implementation of different cervical cancer prevention strategies. Based on the model used for economic evaluation, we performed a budget impact analysis of the different combination strategies which raised concerns on the affordability of implementing both VIA and vaccination in the Philippines (Table 3). Targeting women aged 35–45 years old with VIA is estimated to have a 5-year budget impact of Php 2.8 billion (66.2 million USD). The addition of HPV vaccination for girls 11 years of age on top of VIA screening will increase the financial requirements to Php13.9 billion (328.4 million USD) over five years. This estimated cost excludes other financial requirements of the vaccination program such as vaccine supplies, additional cold chain, funds for training and vaccination campaigns and daily allowances for health providers administering the vaccines not completely incorporated in our analysis for lack of local data. The adoption of HPV vaccination into the national cervical cancer control program will therefore be financially and logistically challenging and will require the government to mobilize additional budgets that will ensure its effective implementation at high coverage.

**Table 3:** Budget impact of optimal choices for cervical cancer prevention in the Philippines

Options	TARGET POPULATION (5-yearly)			5 YEARS BUDGET in million **Php** (USD)				ANNUAL BUDGET in million **Php** (USD)
	VIA	Vaccination (3-dose)	Pap Smear	VIA	Vaccination (3-dose)	Pap Smear	Total	
8 % Pap (35–55)	-	-	941,758	-	-	471 (11.13)	909 (21.48)	182 (4.30)
8 % VIA (35–55)	941,758	-	-	94 (2.22)	-	-	471 (11.13)	94 (2.22)
80 % VIA (35–45)	5,597,648	-	-	560 (13.23)	-	-	2,799 (66.13)	560 (13.23)
80 % VIA (35–50)	7,663,312	-	-	766 (18.10)	-	-	3,832 (90.54)	766 (18.10)
80 % VIA (35–55)	9,417,584	-	-	942 (22.26)	-	-	4,709 (111.26)	942 (22.26)
80 % Vac 11 + 80 % VIA (35–45)	5,597,648	4,059,120	-	560 (13.23)	11,106 (262.40)	-	13,905 (328.53)	2,781 (65.71)
80 % Vac 11 + 80 % VIA (35–50)	7,663,312	4,059,120	-	766 (18.10)	11,106 (262.40)	-	14,937 (352.92)	2,987 (70.57)
80 % Vac 11 + 80 % VIA (35–55)	9,417,584	4,059,120	-	942 (22.26)	11,106 (262.40)	-	15,815 (373.66)	3,163 (74.73)
80 % Vac 11 + 80 % VIA (30–55)	15,704,480	4,059,120	-	1570 (37.09)	11,106 (262.40)	-	18,958 (447.92)	3,792 (89.95)

Our study has some limitations. First, we did not incorporate direct non-medical costs associated with the different preventive strategies such as transportation costs, costs of informal care etc. Second, our analysis did not distinguish between the two existing vaccines, which may differ in several aspects including their potential clinical benefits apart from the prevention of cervical cancer. For example, we did not consider cross-protection which may favor the bivalent vaccine because of its potential greater protective efficacy against non-vaccine oncogenic HPV types particularly against HPV 45 also found to be a common cause of ICC in the Philippines [12, 14, 49, 50]. There is less certainty, however, in the duration of the cross–protective efficacy of both vaccines and its public health significance given that many women with cervical disease have co-infection with vaccine-targeted and non-vaccine HPV types [14, 50].

Third, we referred to the Thai value set for health-related quality of life measures in the absence of disease weights for cervical cancer in the Philippines. While there may be differences between the Thai and Filipino patients in the valuation of health states because of various factors (i.e., ethnicity, cultural perceptions of disease, health system, social support) that may affect the disease weights, this approach was deemed acceptable because of a similar Asian context. However, the need for future triangulation and validation of the results using local utility values in the Philippines is recommended in future analysis.

Lastly, we did not incorporate other clinical endpoints for which the quadrivalent vaccine show protective efficacy such as genital warts, respiratory papillomatoses and precursor lesions of other less common HPV-linked cancers (i.e., anal, vulvar, vaginal cancers) [51, 52]. This would have decreased the cost-effectiveness ratio in favour of the quadrivalent vaccine although we recognize that the most important clinical benefit of both vaccines is in their potential to protect against cervical cancer. These additional endpoints may be of important consideration to Filipino decision-makers when making a choice between the two competing vaccines. Apart from vaccine price, the choice between the vaccines may depend on the preference of Filipino decision-makers on whether they favour the demonstrated strong protection of the quadrivalent vaccine against anogenital warts and non-cervical lesions or the public health potential of the bivalent vaccine to further reduce the incidence of cervical cancer.

## Conclusion

Our analysis shows that the strategy of expanding the coverage of VIA targeting 80 % of adult women 35 to 45 years old done at five-year intervals is the most efficient and cost-saving strategy to implement in the Philippines. Adding a vaccination program among 11-year-old girls at a cost of 54 USD per vaccinated child is potentially cost-effective using the 1 GDP per capita threshold in the Philippine setting. The combination strategy can further reduce cervical cancer burden by two-thirds with the most favourable assumption that the vaccines provide lifelong immunity against HPV 16/18. Other considerations of Philippine policy-makers on decisions about implementing optimal screening and vaccination policies include budget impact, the organization of health services and social acceptability in the local setting.

## Additional files

Additional file 1:
**Distribution HPV types among Filipino women with normal cytology and with invasive cervical cancer.** The figure shows the percentage share of different HPV types among Filipino women with normal cytology and with invasive cervical cancer. Additional file 2:
**Cost and health outcomes of competing strategies at different scenarios and screening 2-A: 8 % screening and 20 % vaccinationcoveragescoverage scenario 2-B: at 80 % screening and 20 % vaccination coveragescoverage scenario 2-C: 8 % screening and 80 % vaccination coveragescoverage scenario 2-D: 80 % screening and 80 % vaccination coveragescoverage scenario.** For each coverage scenario, we identified optimal approaches defined as those having the lowest incremental cost-effectiveness ratios (ICERs) calculated as the additional cost of the incremental benefit of one strategy compared to the next less costly strategy. Additional file 3:
**Cost-effectiveness of HPV vaccination at different start ages of vaccination and frequency of booster doses**. The figure shows the different ICERs achieved with different assumptions on introducing vaccination starting at 11, 13, 20, and 25 years old and with varying frequency of booster doses every 0, 10, 15 and 20- years. Additional file 4:
**Vaccine ceiling price per dose at varying frequency of booster doses and duration of protection.** The table identifies the cost-effective price per dose of the vaccine at varying frequency of booster doses and duration of protection. Additional file 5:
**One-way sensitivity analysis.** The tornado plot describes influential parameters that significantly affect changes in ICERs at 80 % coverage of VIA at 35–55 years old done every five years versus 80 % VIA at 35–55 years old done every five years and 20 % vaccination coverage at11 years old with lifetime protection.
