# Supplementary figures and images for: A cost-utility analysis of cervical cancer screening and human papillomavirus vaccination in the Philippines
Source: BMC Public Health. 2015 Jul 30;15:730. doi: 10.1186/s12889-015-2046-1 (PMC4520072; doi:10.1186/s12889-015-2046-1)

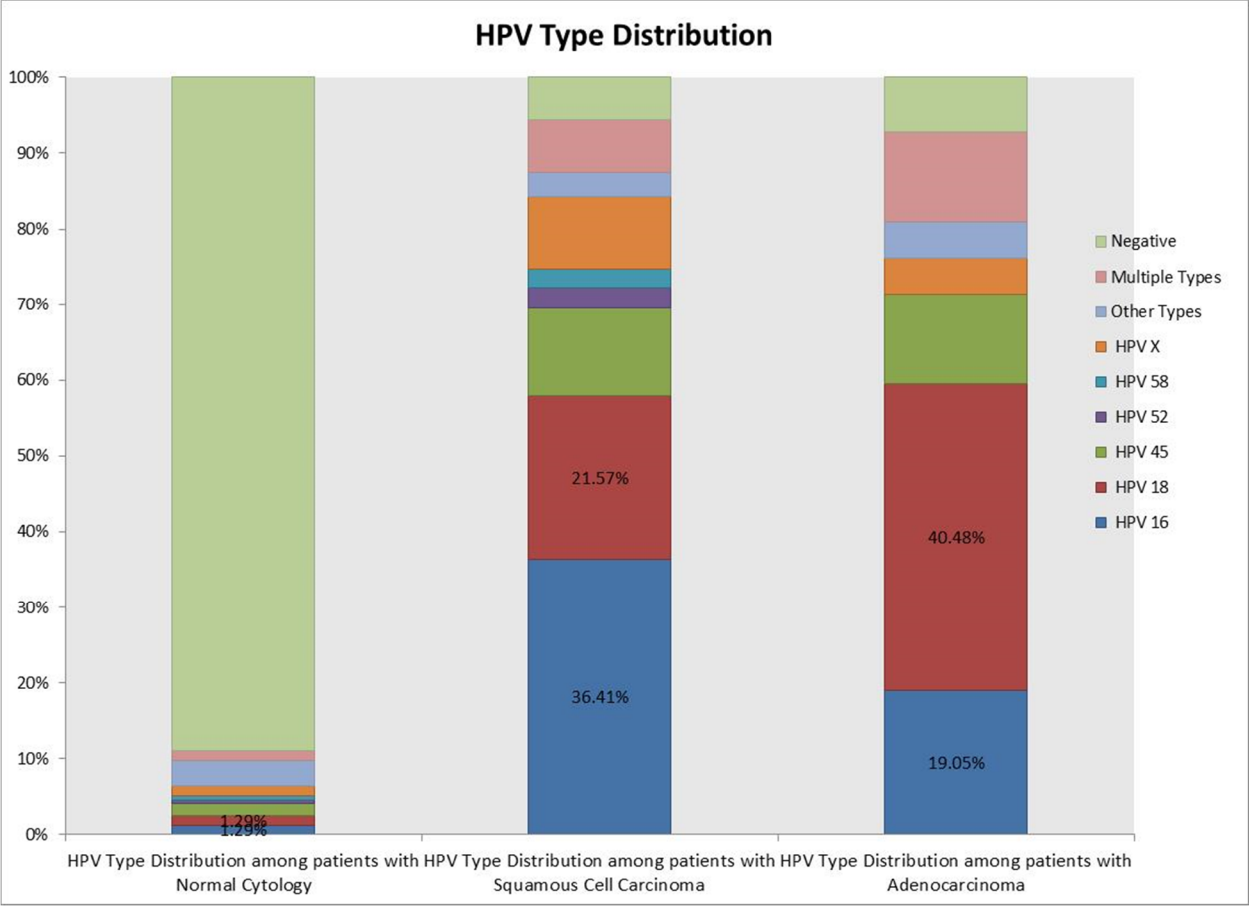

Supplement: Additional file 1: — Distribution HPV types among Filipino women with normal cytology and with invasive cervical cancer. The figure shows the percentage share of different HPV types among Filipino women with normal cytology and with invasive cervical cancer. [file 12889_2015_2046_MOESM1_ESM.pdf]

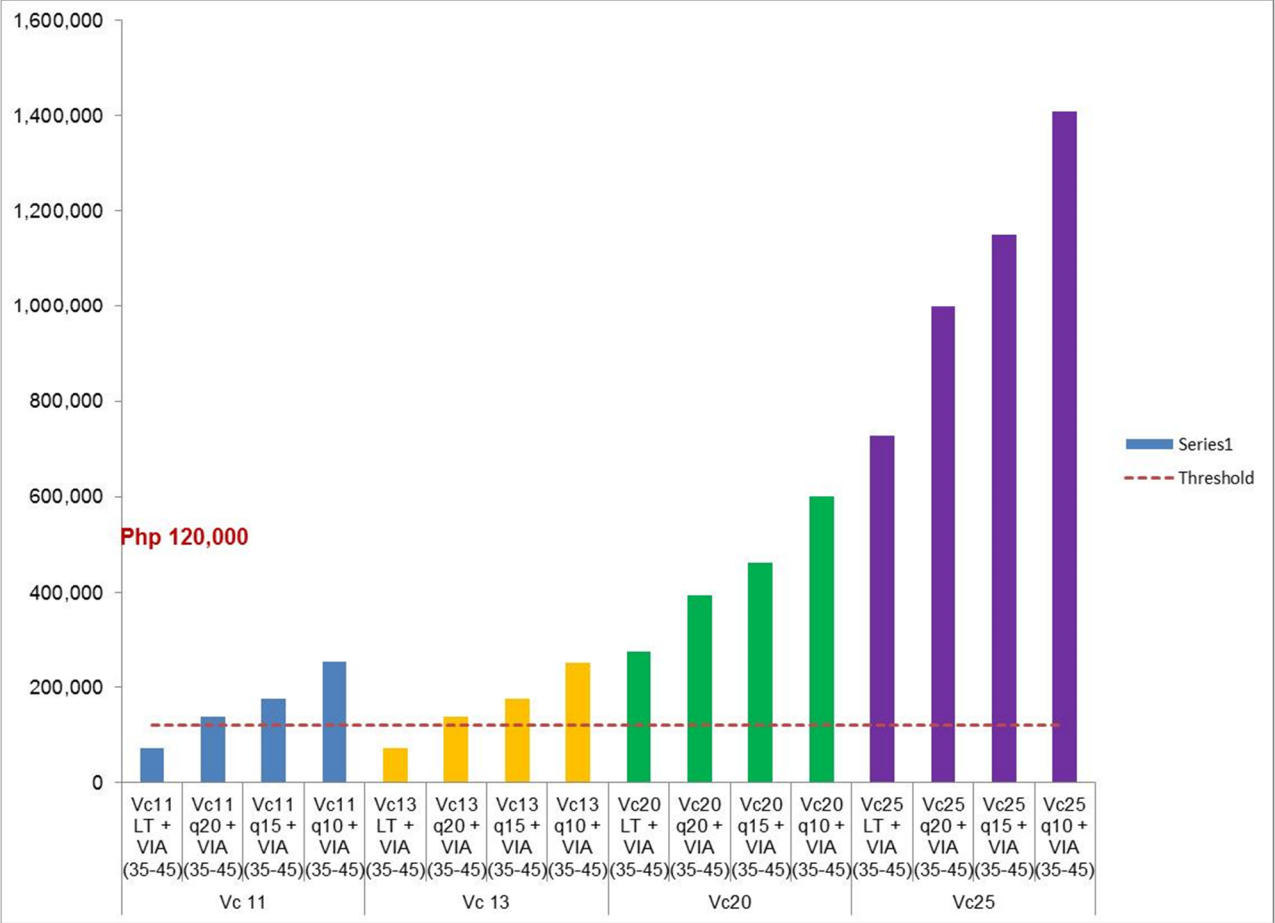

Supplement: Additional file 3: — Cost-effectiveness of HPV vaccination at different start ages of vaccination and frequency of booster doses. The figure shows the different ICERs achieved with different assumptions on introducing vaccination starting at 11, 13, 20, and 25 years old and with varying frequency of booster doses every 0, 10, 15 and 20- years. [file 12889_2015_2046_MOESM3_ESM.pdf]

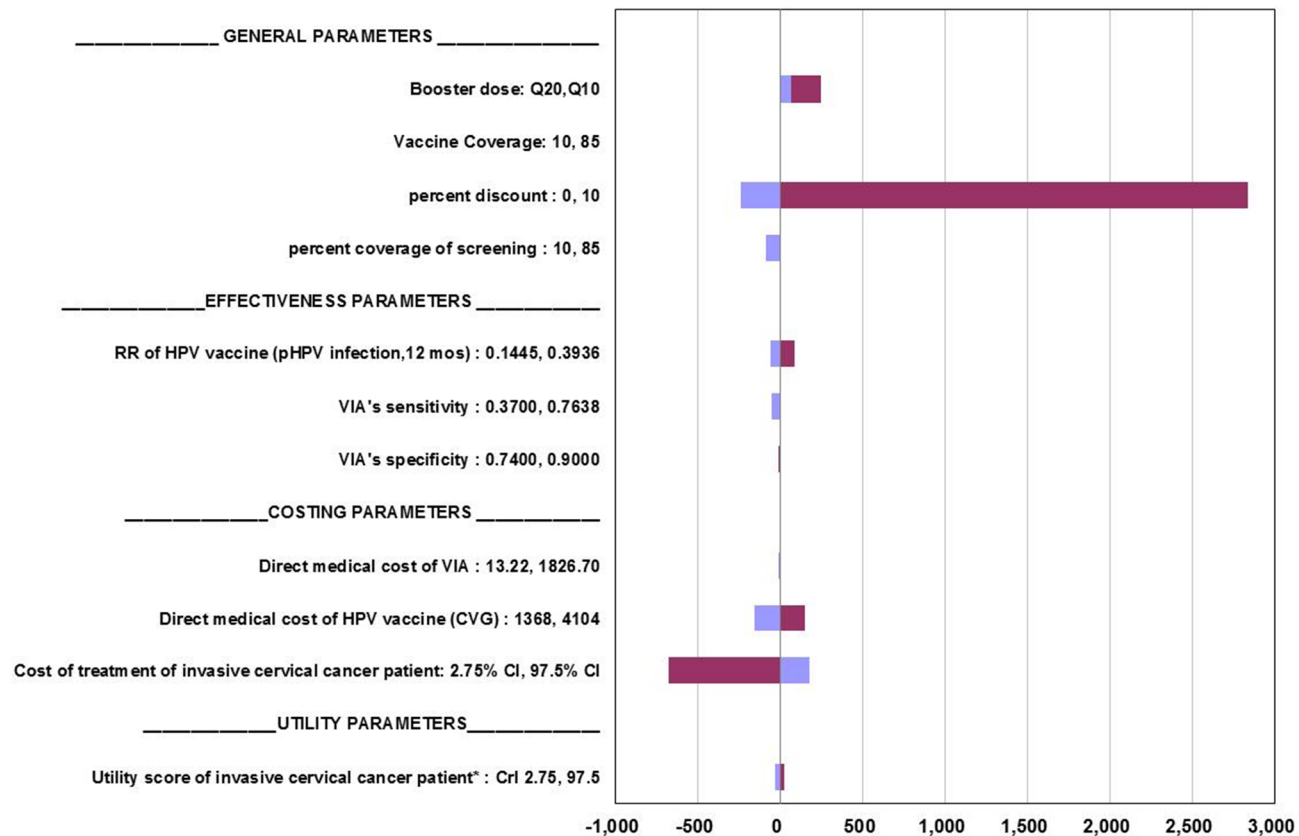

Supplement: Additional file 5: — One-way sensitivity analysis. The tornado plot describes influential parameters that significantly affect changes in ICERs at 80 % coverage of VIA at 35–55 years old done every five years versus 80 % VIA at 35–55 years old done every five years and 20 % vaccination coverage at11 years old with lifetime protection. [file 12889_2015_2046_MOESM5_ESM.pdf]
