# Supplementary material for: A cost-utility analysis of cervical cancer screening and human papillomavirus vaccination in the Philippines
Source: BMC Public Health. 2015 Jul 30;15:730. doi: 10.1186/s12889-015-2046-1 (PMC4520072; doi:10.1186/s12889-015-2046-1)
Supplement: Additional file 4: — Vaccine ceiling price per dose at varying frequency of booster doses and duration of protection. The table identifies the cost-effective price per dose of the vaccine at varying frequency of booster doses and duration of protection. [file 12889_2015_2046_MOESM4_ESM.docx]

Table 1*.* Vaccine ceiling price per dose at varying frequency of booster doses and duration of protection

|  | Policy Options | WTP at 1 GDP per capita (Php) | WTP at 0  (Php) |
| --- | --- | --- | --- |
| 1 booster dose | Vc11 LT + VIA (35-45) | 1050.49 | 701.67 |
|  | Vc11 q20 + VIA (35-45) | 867.23 | 579.26 |
|  | Vc11 q15 + VIA (35-45) | 790.17 | 527.79 |
|  | Vc11 q10 + VIA (35-45) | 667.14 | 445.61 |
| 2 booster doses | Vc11 LT + VIA (35-45) | 1050.49 | 701.67 |
|  | Vc11 q20 + VIA (35-45) | 738.41 | 493.22 |
|  | Vc11 q15 + VIA (35-45) | 633.25 | 422.97 |
|  | Vc11 q10 + VIA (35-45) | 488.77 | 326.47 |
| 3 booster doses | Vc11 LT + VIA (35-45) | 1050.49 | 701.67 |
|  | Vc11 q20 + VIA (35-45) | 642.92 | 429.43 |
|  | Vc11 q15 + VIA (35-45) | 528.33 | 352.89 |
|  | Vc11 q10 + VIA (35-45) | 385.66 | 257.60 |

Table 2-A. Cost and health outcomes of competing strategies at 8% screening and 20% vaccination coverages

|  | Strategies | Cost | | | | LY s | LE | QALYs | | | | ICER |
| --- | --- | --- | --- | --- | --- | --- | --- | --- | --- | --- | --- | --- |
|  |  | Total | prevention | | treatment of  cervical cancer |  |  | Total | Healthy | HPV infection - CIN | cervical cancer |  |
|  |  |  | vaccination | CIN |  |  |  |  |  |  |  |  |
| 1 | Pap q5y (20 - 55) | 4,087 | 0 | 0 | 4,087 | 25.88600 | 65.71 | 25.87236 | 24.48558 | 1.342303 | 0.044477 | Dom |
| 2 | VIA q5Y (35-45) | 4,059 | 0 | 92 | 3,967 | 25.88628 | 68.46 | 25.87306 | 24.48984 | 1.340040 | 0.043183 | Dom |
| 3 | Pap q5y (35 - 55) | 4,089 | 0 | 133 | 3,956 | 25.88632 | 70.22 | 25.87315 | 24.49047 | 1.339622 | 0.043057 | Reference |
| 4 | Pap q5y (35 - 60) | 4,094 | 0 | 147 | 3,947 | 25.88635 | 71.05 | 25.87321 | 24.49092 | 1.339340 | 0.042950 | Dom |
| 5 | VIA q5Y (30-45) | 4,073 | 0 | 135 | 3,938 | 25.88635 | 69.38 | 25.87324 | 24.49084 | 1.339546 | 0.042855 | Dom |
| 6 | VIA q5Y (35-50) | 4,050 | 0 | 112 | 3,938 | 25.88636 | 69.35 | 25.87324 | 24.49102 | 1.339366 | 0.042860 | Dom |
| 7 | Pap q5y (35 - 65) | 4,101 | 0 | 158 | 3,943 | 25.88636 | 71.84 | 25.87324 | 24.49120 | 1.339158 | 0.042890 | Dom |
| 8 | Pap q5y (30 - 55) | 4,111 | 0 | 177 | 3,933 | 25.88637 | 71.14 | 25.87329 | 24.49125 | 1.339239 | 0.042804 | Dom |
| 9 | Pap q5y (30 - 60) | 4,116 | 0 | 191 | 3,924 | 25.88640 | 71.98 | 25.87335 | 24.49169 | 1.338958 | 0.042697 | Dom |
| 10 | VIA q5Y (35-45) + Pap q5y (50 - 60) | 4,065 | 0 | 145 | 3,921 | 25.88641 | 71.06 | 25.87336 | 24.49186 | 1.338840 | 0.042658 | Dom |
| 11 | VIA q5Y (35-55) | 4,048 | 0 | 129 | 3,918 | 25.88641 | 70.22 | 25.87337 | 24.49188 | 1.338851 | 0.042638 | -191,099.19 |
| 12 | Vac 25Y boost q10 | 4,398 | 499 | 0 | 3,899 | 25.88641 | 65.72 | 25.87338 | 24.52936 | 1.301552 | 0.042473 | Dom |
| 13 | Vac 25Y boost q15 | 4,332 | 433 | 0 | 3,899 | 25.88641 | 65.72 | 25.87338 | 24.52936 | 1.301552 | 0.042473 | Dom |
| 14 | Vac 25Y boost q20 | 4,295 | 396 | 0 | 3,899 | 25.88641 | 65.72 | 25.87338 | 24.52936 | 1.301552 | 0.042473 | Dom |
| 15 | Vac 25Y LT | 4,227 | 328 | 0 | 3,899 | 25.88641 | 65.72 | 25.87338 | 24.52936 | 1.301552 | 0.042473 | Dom |
| 16 | Pap q5y (30 - 65) | 4,122 | 0 | 202 | 3,920 | 25.88642 | 72.76 | 25.87338 | 24.49197 | 1.338775 | 0.042637 | Dom |
| 17 | VIA q5Y (35-45) + Pap q5y (50 - 65) | 4,072 | 0 | 156 | 3,916 | 25.88643 | 71.84 | 25.87340 | 24.49214 | 1.338657 | 0.042598 | Dom |
| 18 | VIA q5Y (35-50) + Pap q5y (55 - 60) | 4,058 | 0 | 144 | 3,914 | 25.88642 | 71.06 | 25.87340 | 24.49213 | 1.338690 | 0.042584 | Dom |
| 19 | VIA q5Y (25-45) | 4,095 | 0 | 186 | 3,909 | 25.88642 | 70.31 | 25.87342 | 24.49179 | 1.339101 | 0.042529 | Dom |
| 20 | VIA q5Y (30-50) | 4,064 | 0 | 156 | 3,908 | 25.88643 | 70.27 | 25.87342 | 24.49201 | 1.338874 | 0.042533 | Dom |
| 21 | Pap q5y (25 - 55) | 4,141 | 0 | 230 | 3,911 | 25.88643 | 72.07 | 25.87342 | 24.49198 | 1.338893 | 0.042551 | Dom |
| 22 | VIA q5Y (35-50) + Pap q5y (55 - 65) | 4,065 | 0 | 155 | 3,909 | 25.88644 | 71.84 | 25.87343 | 24.49240 | 1.338508 | 0.042524 | Dom |
| 23 | VIA q5Y (35-55) + Pap q5y (60 - 65) | 4,059 | 0 | 155 | 3,905 | 25.88645 | 71.84 | 25.87346 | 24.49260 | 1.338393 | 0.042473 | Dom |
| 24 | Pap q5y (25 - 60) | 4,146 | 0 | 244 | 3,902 | 25.88645 | 72.90 | 25.87349 | 24.49243 | 1.338611 | 0.042444 | Dom |
| 25 | VIA q5Y (20-45) | 4,143 | 0 | 248 | 3,895 | 25.88645 | 71.20 | 25.87350 | 24.49223 | 1.338902 | 0.042371 | Dom |
| 26 | Pap q5y (25 - 65) | 4,153 | 0 | 255 | 3,897 | 25.88647 | 73.69 | 25.87352 | 24.49271 | 1.338429 | 0.042384 | Dom |
| 27 | VIA q5Y (30-45) + Pap q5y (50 - 60) | 4,079 | 0 | 188 | 3,891 | 25.88648 | 71.98 | 25.87354 | 24.49286 | 1.338348 | 0.042331 | Dom |
| 28 | VIA q5Y (30-55) | 4,061 | 0 | 173 | 3,889 | 25.88648 | 71.14 | 25.87354 | 24.49288 | 1.338359 | 0.042311 | Dom |
| 29 | Pap q5y (20 - 60) | 4,198 | 0 | 307 | 3,891 | 25.88648 | 73.79 | 25.87355 | 24.49277 | 1.338456 | 0.042321 | Dom |
| 30 | VIA q5Y (30-45) + Pap q5y (50 - 65) | 4,085 | 0 | 199 | 3,887 | 25.88650 | 72.76 | 25.87357 | 24.49314 | 1.338166 | 0.042271 | Dom |
| 31 | VIA q5Y (30-50) + Pap q5y (55 - 60) | 4,072 | 0 | 187 | 3,885 | 25.88650 | 71.98 | 25.87358 | 24.49312 | 1.338198 | 0.042257 | Dom |
| 32 | Pap q5y (20 - 65) | 4,205 | 0 | 318 | 3,887 | 25.88650 | 74.58 | 25.87358 | 24.49305 | 1.338273 | 0.042260 | Dom |
| 33 | VIA q5Y (25-50) | 4,087 | 0 | 207 | 3,880 | 25.88650 | 71.20 | 25.87359 | 24.49296 | 1.338430 | 0.042207 | Dom |
| 34 | VIA q5Y (30-50) + Pap q5y (55 - 65) | 4,078 | 0 | 198 | 3,880 | 25.88651 | 72.76 | 25.87361 | 24.49340 | 1.338017 | 0.042197 | Dom |
| 35 | VIA q5Y (30-55) + Pap q5y (60 - 65) | 4,073 | 0 | 198 | 3,875 | 25.88652 | 72.77 | 25.87364 | 24.49359 | 1.337902 | 0.042146 | Dom |
| 36 | VIA q5Y (20-50) | 4,134 | 0 | 268 | 3,866 | 25.88653 | 72.09 | 25.87368 | 24.49340 | 1.338230 | 0.042048 | Dom |
| 37 | VIA q5Y (25-45) + Pap q5y (50 - 60) | 4,102 | 0 | 239 | 3,862 | 25.88655 | 72.90 | 25.87371 | 24.49380 | 1.337904 | 0.042005 | Dom |
| 38 | VIA q5Y (25-55) | 4,084 | 0 | 224 | 3,860 | 25.88655 | 72.07 | 25.87372 | 24.49382 | 1.337915 | 0.041985 | Dom |
| 39 | VIA q5Y (25-45) + Pap q5y (50 - 65) | 4,108 | 0 | 250 | 3,858 | 25.88656 | 73.69 | 25.87375 | 24.49408 | 1.337722 | 0.041945 | Dom |
| 40 | VIA q5Y (25-50) + Pap q5y (55 - 65) | 4,101 | 0 | 250 | 3,851 | 25.88658 | 73.69 | 25.87379 | 24.49434 | 1.337572 | 0.041871 | Dom |
| 41 | VIA q5Y (20-45) + Pap q5y (50 - 60) | 4,149 | 0 | 301 | 3,849 | 25.88658 | 73.79 | 25.87379 | 24.49424 | 1.337704 | 0.041847 | Dom |
| 42 | VIA q5Y (20-55) | 4,132 | 0 | 285 | 3,846 | 25.88658 | 72.96 | 25.87380 | 24.49426 | 1.337716 | 0.041826 | Dom |
| 43 | VIA q5Y (25-55) + Pap q5y (60 - 65) | 4,096 | 0 | 249 | 3,847 | 25.88659 | 73.69 | 25.87381 | 24.49453 | 1.337458 | 0.041820 | Dom |
| 44 | VIA q5Y (20-45) + Pap q5y (50 - 65) | 4,156 | 0 | 312 | 3,844 | 25.88660 | 74.58 | 25.87383 | 24.49452 | 1.337522 | 0.041786 | Dom |
| 45 | VIA q5Y (20-50) + Pap q5y (55 - 60) | 4,142 | 0 | 300 | 3,842 | 25.88660 | 73.79 | 25.87383 | 24.49451 | 1.337555 | 0.041773 | Dom |
| 46 | VIA q5Y (20-50) + Pap q5y (55 - 60) | 4,142 | 0 | 300 | 3,842 | 25.88660 | 73.79 | 25.87383 | 24.49451 | 1.337555 | 0.041773 | Dom |
| 47 | VIA q5Y (20-50) + Pap q5y (55 - 65) | 4,148 | 0 | 311 | 3,838 | 25.88661 | 74.58 | 25.87387 | 24.49478 | 1.337373 | 0.041713 | Dom |
| 48 | VIA q5Y (20-55) + Pap q5y (60 - 65) | 4,143 | 0 | 310 | 3,833 | 25.88662 | 74.58 | 25.87389 | 24.49497 | 1.337258 | 0.041661 | Dom |
| 49 | Vac 20Y boost q10 | 4,385 | 578 | 0 | 3,807 | 25.88662 | 65.72 | 25.87390 | 24.56278 | 1.269636 | 0.041481 | Dom |
| 50 | Vac 20Y boost q15 | 4,301 | 494 | 0 | 3,807 | 25.88662 | 65.72 | 25.87390 | 24.56278 | 1.269636 | 0.041481 | Dom |
| 51 | Vac 20Y boost q20 | 4,260 | 453 | 0 | 3,807 | 25.88662 | 65.72 | 25.87390 | 24.56278 | 1.269636 | 0.041481 | Dom |
| 52 | Vac 20Y LT | 4,186 | 379 | 0 | 3,807 | 25.88662 | 65.72 | 25.87390 | 24.56278 | 1.269636 | 0.041481 | Dom |
| 53 | Vac 13Y boost q10 | 4,382 | 793 | 0 | 3,589 | 25.88713 | 65.72 | 25.87515 | 24.65744 | 1.178645 | 0.039065 | Dom |
| 54 | Vac 13Y boost q15 | 4,259 | 671 | 0 | 3,589 | 25.88713 | 65.72 | 25.87515 | 24.65744 | 1.178645 | 0.039065 | Dom |
| 55 | Vac 13Y boost q20 | 4,200 | 611 | 0 | 3,589 | 25.88713 | 65.72 | 25.87515 | 24.65744 | 1.178645 | 0.039065 | Dom |
| 56 | Vac 13Y LT | 4,093 | 505 | 0 | 3,588 | 25.88713 | 65.72 | 25.87515 | 24.65744 | 1.178645 | 0.039065 | Dom |
| 57 | Vac 12Y boost q10 | 4,405 | 834 | 0 | 3,571 | 25.88717 | 65.72 | 25.87525 | 24.66543 | 1.170943 | 0.038871 | Dom |
| 58 | Vac 12Y boost q15 | 4,275 | 703 | 0 | 3,571 | 25.88717 | 65.72 | 25.87525 | 24.66543 | 1.170943 | 0.038871 | Dom |
| 59 | Vac 12Y boost q20 | 4,212 | 640 | 0 | 3,571 | 25.88717 | 65.72 | 25.87525 | 24.66543 | 1.170943 | 0.038871 | Dom |
| 60 | Vac 12Y LT | 4,100 | 528 | 0 | 3,571 | 25.88717 | 65.72 | 25.87525 | 24.66543 | 1.170943 | 0.038871 | Dom |
| 61 | Vac 11Y boost q10 | 4,424 | 871 | 0 | 3,553 | 25.88721 | 65.72 | 25.87535 | 24.67390 | 1.162788 | 0.038666 | Dom |
| 62 | Vac 11Y boost q15 | 4,285 | 732 | 0 | 3,553 | 25.88721 | 65.72 | 25.87535 | 24.67390 | 1.162788 | 0.038666 | Dom |
| 63 | Vac 11Y boost q20 | 4,219 | 665 | 0 | 3,553 | 25.88721 | 65.72 | 25.87535 | 24.67390 | 1.162788 | 0.038666 | Dom |
| 64 | Vac 11Y LT | 4,100 | 547 | 0 | 3,553 | 25.88721 | 65.72 | 25.87535 | 24.67390 | 1.162788 | 0.038666 | Dom |
| 65 | Vac 11Y LT + VIA q5Y (35-45) | 4,088 | 547 | 91 | 3,450 | 25.88745 | 68.49 | 25.87596 | 24.67760 | 1.160821 | 0.037541 | Dom |
| 66 | Vac 11Y LT + Pap q5y (35 - 55) | 4,119 | 547 | 133 | 3,440 | 25.88748 | 70.26 | 25.87604 | 24.67815 | 1.160455 | 0.037430 | Dom |
| 67 | Vac 11Y LT + Pap q5y (35 - 60) | 4,126 | 547 | 146 | 3,432 | 25.88751 | 71.09 | 25.87609 | 24.67855 | 1.160209 | 0.037337 | Dom |
| 68 | Vac 11Y LT + VIA q5Y (30-45) | 4,105 | 547 | 134 | 3,424 | 25.88751 | 69.42 | 25.87612 | 24.67846 | 1.160395 | 0.037258 | Dom |
| 69 | Vac 11Y LT + VIA q5Y (35-50) | 4,083 | 547 | 112 | 3,424 | 25.88752 | 69.38 | 25.87612 | 24.67863 | 1.160234 | 0.037259 | Dom |
| 70 | Vac 11Y LT + Pap q5y (35 - 65) | 4,133 | 547 | 157 | 3,428 | 25.88752 | 71.88 | 25.87612 | 24.67879 | 1.160049 | 0.037284 | Dom |
| 71 | Vac 11Y LT + Pap q5y (30 - 55) | 4,144 | 547 | 177 | 3,420 | 25.88753 | 71.19 | 25.87616 | 24.67882 | 1.160125 | 0.037211 | Dom |
| 72 | Vac 11Y LT + Pap q5y (30 - 60) | 4,150 | 547 | 191 | 3,412 | 25.88755 | 72.03 | 25.87621 | 24.67922 | 1.159879 | 0.037118 | Dom |
| 73 | Vac 11Y LT + VIA q5Y (35-45) + Pap q5y (50 - 60) | 4,100 | 547 | 144 | 3,409 | 25.88756 | 71.10 | 25.87622 | 24.67937 | 1.159774 | 0.037083 | Dom |
| 74 | Vac 11Y LT + VIA q5Y (35-55) | 4,083 | 547 | 129 | 3,407 | 25.88756 | 70.26 | 25.87623 | 24.67938 | 1.159784 | 0.037065 | Dom |
| 75 | Vac 11Y LT + Pap q5y (30 - 65) | 4,157 | 547 | 202 | 3,408 | 25.88757 | 72.82 | 25.87624 | 24.67946 | 1.159719 | 0.037065 | Dom |
| 76 | Vac 11Y LT + VIA q5Y (35-45) + Pap q5y (50 - 65) | 4,107 | 547 | 155 | 3,405 | 25.88758 | 71.89 | 25.87625 | 24.67961 | 1.159614 | 0.037030 | Dom |
| 77 | Vac 11Y LT + VIA q5Y (35-50) + Pap q5y (55 - 60) | 4,093 | 547 | 143 | 3,403 | 25.88758 | 71.10 | 25.87626 | 24.67960 | 1.159643 | 0.037018 | Dom |
| 78 | Vac 11Y LT + VIA q5Y (25-45) | 4,132 | 547 | 185 | 3,399 | 25.88757 | 70.35 | 25.87627 | 24.67928 | 1.160013 | 0.036978 | Dom |
| 79 | Vac 11Y LT + VIA q5Y (30-50) | 4,100 | 547 | 155 | 3,398 | 25.88758 | 70.31 | 25.87627 | 24.67949 | 1.159809 | 0.036977 | Dom |
| 80 | Vac 11Y LT + Pap q5y (25 - 55) | 4,177 | 547 | 229 | 3,401 | 25.88758 | 72.12 | 25.87628 | 24.67945 | 1.159827 | 0.036994 | Dom |
| 81 | Vac 11Y LT + VIA q5Y (35-50) + Pap q5y (55 - 65) | 4,100 | 547 | 154 | 3,399 | 25.88759 | 71.89 | 25.87629 | 24.67984 | 1.159484 | 0.036965 | Dom |
| 82 | Vac 11Y LT + VIA q5Y (35-55) + Pap q5y (60 - 65) | 4,096 | 547 | 154 | 3,395 | 25.88760 | 71.89 | 25.87631 | 24.68001 | 1.159384 | 0.036921 | Dom |
| 83 | Vac 11Y LT + Pap q5y (25 - 60) | 4,183 | 547 | 243 | 3,393 | 25.88760 | 72.96 | 25.87633 | 24.67985 | 1.159581 | 0.036901 | Dom |
| 84 | Vac 11Y LT + Pap q5y (20 - 55) | 4,231 | 547 | 292 | 3,392 | 25.88760 | 73.02 | 25.87633 | 24.67975 | 1.159694 | 0.036889 | Dom |
| 85 | Vac 11Y LT + VIA q5Y (20-45) | 4,181 | 547 | 246 | 3,388 | 25.88760 | 71.25 | 25.87634 | 24.67965 | 1.159842 | 0.036842 | Dom |
| 86 | Vac 11Y LT + Pap q5y (25 - 65) | 4,190 | 547 | 254 | 3,389 | 25.88762 | 73.75 | 25.87636 | 24.68009 | 1.159422 | 0.036848 | Dom |
| 87 | Vac 11Y LT + VIA q5Y (30-45) + Pap q5y (50 - 60) | 4,117 | 547 | 187 | 3,383 | 25.88762 | 72.03 | 25.87638 | 24.68023 | 1.159350 | 0.036801 | Dom |
| 88 | Vac 11Y LT + VIA q5Y (30-55) | 4,100 | 547 | 172 | 3,381 | 25.88762 | 71.19 | 25.87638 | 24.68024 | 1.159360 | 0.036783 | Dom |
| 89 | Vac 11Y LT + Pap q5y (20 - 60) | 4,237 | 547 | 306 | 3,384 | 25.88762 | 73.86 | 25.87638 | 24.68014 | 1.159448 | 0.036796 | Dom |
| 90 | Vac 11Y LT + VIA q5Y (30-45) + Pap q5y (50 - 65) | 4,124 | 547 | 198 | 3,379 | 25.88764 | 72.82 | 25.87641 | 24.68047 | 1.159190 | 0.036748 | Dom |
| 91 | Vac 11Y LT + VIA q5Y (30-50) + Pap q5y (55 - 60) | 4,111 | 547 | 186 | 3,377 | 25.88764 | 72.03 | 25.87641 | 24.68046 | 1.159219 | 0.036736 | Dom |
| 92 | Vac 11Y LT + Pap q5y (20 - 65) | 4,244 | 547 | 317 | 3,380 | 25.88764 | 74.65 | 25.87641 | 24.68038 | 1.159289 | 0.036743 | Dom |
| 93 | Vac 11Y LT + VIA q5Y (25-50) | 4,127 | 547 | 206 | 3,374 | 25.88764 | 71.25 | 25.87642 | 24.68030 | 1.159427 | 0.036697 | Dom |
| 94 | Vac 11Y LT + VIA q5Y (30-50) + Pap q5y (55 - 65) | 4,118 | 547 | 197 | 3,374 | 25.88765 | 72.82 | 25.87644 | 24.68070 | 1.159060 | 0.036684 | Dom |
| 95 | Vac 11Y LT + VIA q5Y (30-55) + Pap q5y (60 - 65) | 4,113 | 547 | 197 | 3,370 | 25.88766 | 72.82 | 25.87646 | 24.68086 | 1.158960 | 0.036639 | Dom |
| 96 | Vac 11Y LT + VIA q5Y (20-50) | 4,176 | 547 | 267 | 3,362 | 25.88766 | 72.15 | 25.87649 | 24.68067 | 1.159257 | 0.036561 | Dom |
| 97 | Vac 11Y LT + VIA q5Y (25-45) + Pap q5y (50 - 60) | 4,144 | 547 | 238 | 3,359 | 25.88768 | 72.96 | 25.87653 | 24.68104 | 1.158968 | 0.036521 | Dom |
| 98 | Vac 11Y LT + VIA q5Y (25-55) | 4,127 | 547 | 223 | 3,356 | 25.88768 | 72.12 | 25.87653 | 24.68105 | 1.158978 | 0.036503 | Dom |
| 99 | Vac 11Y LT + VIA q5Y (25-45) + Pap q5y (50 - 65) | 4,151 | 547 | 249 | 3,355 | 25.88770 | 73.75 | 25.87656 | 24.68128 | 1.158809 | 0.036468 | Dom |
| 100 | Vac 11Y LT + VIA q5Y (25-50) + Pap q5y (55 - 65) | 4,145 | 547 | 249 | 3,349 | 25.88771 | 73.75 | 25.87659 | 24.68151 | 1.158679 | 0.036404 | Dom |
| 101 | Vac 11Y LT + VIA q5Y (20-45) + Pap q5y (50 - 60) | 4,193 | 547 | 299 | 3,347 | 25.88771 | 73.86 | 25.87660 | 24.68141 | 1.158798 | 0.036385 | Dom |
| 102 | Vac 11Y LT + VIA q5Y (20-55) | 4,176 | 547 | 284 | 3,345 | 25.88771 | 73.03 | 25.87660 | 24.68142 | 1.158808 | 0.036367 | Dom |
| 103 | Vac 11Y LT + VIA q5Y (25-55) + Pap q5y (60 - 65) | 4,140 | 547 | 248 | 3,345 | 25.88772 | 73.75 | 25.87661 | 24.68167 | 1.158578 | 0.036359 | Dom |
| 104 | Vac 11Y LT + VIA q5Y (20-45) + Pap q5y (50 - 65) | 4,200 | 547 | 310 | 3,343 | 25.88772 | 74.65 | 25.87663 | 24.68165 | 1.158638 | 0.036332 | Dom |
| 105 | Vac 11Y LT + VIA q5Y (20-50) + Pap q5y (55 - 60) | 4,187 | 547 | 298 | 3,341 | 25.88772 | 73.87 | 25.87663 | 24.68164 | 1.158667 | 0.036321 | Dom |
| 106 | Vac 11Y LT + VIA q5Y (20-50) + Pap q5y (55 - 60) | 4,187 | 547 | 298 | 3,341 | 25.88772 | 73.87 | 25.87663 | 24.68164 | 1.158667 | 0.036321 | Dom |
| 107 | Vac 11Y LT + VIA q5Y (20-50) + Pap q5y (55 - 65) | 4,194 | 547 | 309 | 3,337 | 25.88774 | 74.65 | 25.87666 | 24.68188 | 1.158508 | 0.036268 | Dom |
| 108 | Vac 11Y LT + VIA q5Y (20-55) + Pap q5y (60 - 65) | 4,189 | 547 | 309 | 3,333 | 25.88775 | 74.66 | 25.87668 | 24.68205 | 1.158408 | 0.036223 | Dom |

Table 2-B. Cost and health outcomes of competing strategies at 80% screening and 20% vaccination coverages

|  | Strategies | Cost | | | | LY s | LE | QALYs | | | | ICER |
| --- | --- | --- | --- | --- | --- | --- | --- | --- | --- | --- | --- | --- |
|  |  | Total | prevention | | treatment of  cervical cancer |  |  | Total | Healthy | HPV infection - CIN | cervical cancer |  |
|  |  |  | vaccination | CIN |  |  |  |  |  |  |  |  |
| 1 | Pap q5y (20 - 55) | 4,087 | 0 | 0 | 4,087 | 25.88600 | 65.71 | 25.87236 | 24.48558 | 1.34230 | 0.04448 | Dom |
| 2 | Vac 25Y boost q10 | 4,398 | 499 | 0 | 3,899 | 25.88641 | 65.72 | 25.87338 | 24.52936 | 1.30155 | 0.04247 | Dom |
| 3 | Vac 25Y boost q15 | 4,332 | 433 | 0 | 3,899 | 25.88641 | 65.72 | 25.87338 | 24.52936 | 1.30155 | 0.04247 | Dom |
| 4 | Vac 25Y boost q20 | 4,295 | 396 | 0 | 3,899 | 25.88641 | 65.72 | 25.87338 | 24.52936 | 1.30155 | 0.04247 | Dom |
| 5 | Vac 25Y LT | 4,227 | 328 | 0 | 3,899 | 25.88641 | 65.72 | 25.87338 | 24.52936 | 1.30155 | 0.04247 | Dom |
| 6 | Vac 20Y boost q10 | 4,385 | 578 | 0 | 3,807 | 25.88662 | 65.72 | 25.87390 | 24.56278 | 1.26964 | 0.04148 | Dom |
| 7 | Vac 20Y boost q15 | 4,301 | 494 | 0 | 3,807 | 25.88662 | 65.72 | 25.87390 | 24.56278 | 1.26964 | 0.04148 | Dom |
| 8 | Vac 20Y boost q20 | 4,260 | 453 | 0 | 3,807 | 25.88662 | 65.72 | 25.87390 | 24.56278 | 1.26964 | 0.04148 | Dom |
| 9 | Vac 20Y LT | 4,186 | 379 | 0 | 3,807 | 25.88662 | 65.72 | 25.87390 | 24.56278 | 1.26964 | 0.04148 | Dom |
| 10 | Vac 13Y boost q10 | 4,382 | 793 | 0 | 3,589 | 25.88713 | 65.72 | 25.87515 | 24.65744 | 1.17864 | 0.03907 | Dom |
| 11 | Vac 13Y boost q15 | 4,259 | 671 | 0 | 3,589 | 25.88713 | 65.72 | 25.87515 | 24.65744 | 1.17864 | 0.03907 | Dom |
| 12 | Vac 13Y boost q20 | 4,200 | 611 | 0 | 3,589 | 25.88713 | 65.72 | 25.87515 | 24.65744 | 1.17864 | 0.03907 | Dom |
| 13 | Vac 13Y LT | 4,093 | 505 | 0 | 3,588 | 25.88713 | 65.72 | 25.87515 | 24.65744 | 1.17864 | 0.03907 | Dom |
| 14 | Vac 12Y boost q10 | 4,405 | 834 | 0 | 3,571 | 25.88717 | 65.72 | 25.87525 | 24.66543 | 1.17094 | 0.03887 | Dom |
| 15 | Vac 12Y boost q15 | 4,275 | 703 | 0 | 3,571 | 25.88717 | 65.72 | 25.87525 | 24.66543 | 1.17094 | 0.03887 | Dom |
| 16 | Vac 12Y boost q20 | 4,212 | 640 | 0 | 3,571 | 25.88717 | 65.72 | 25.87525 | 24.66543 | 1.17094 | 0.03887 | Dom |
| 17 | Vac 12Y LT | 4,100 | 528 | 0 | 3,571 | 25.88717 | 65.72 | 25.87525 | 24.66543 | 1.17094 | 0.03887 | Dom |
| 18 | Vac 11Y boost q10 | 4,424 | 871 | 0 | 3,553 | 25.88721 | 65.72 | 25.87535 | 24.67390 | 1.16279 | 0.03867 | Dom |
| 19 | Vac 11Y boost q15 | 4,285 | 732 | 0 | 3,553 | 25.88721 | 65.72 | 25.87535 | 24.67390 | 1.16279 | 0.03867 | Dom |
| 20 | Vac 11Y boost q20 | 4,219 | 665 | 0 | 3,553 | 25.88721 | 65.72 | 25.87535 | 24.67390 | 1.16279 | 0.03867 | Dom |
| 21 | Vac 11Y LT | 4,100 | 547 | 0 | 3,553 | 25.88721 | 65.72 | 25.87535 | 24.67390 | 1.16279 | 0.03867 | Dom |
| 22 | VIA q5Y (35-45) | 3,991 | 0 | 912 | 3,079 | 25.88838 | 68.48 | 25.87829 | 24.52144 | 1.32334 | 0.03352 | - 19,189.52 |
| 23 | Pap q5y (35 - 55) | 4,332 | 0 | 1,324 | 3,008 | 25.88859 | 70.25 | 25.87882 | 24.52556 | 1.32054 | 0.03272 | Reference |
| 24 | Pap q5y (35 - 60) | 4,413 | 0 | 1,463 | 2,950 | 25.88876 | 71.09 | 25.87920 | 24.52842 | 1.31874 | 0.03203 | Dom |
| 25 | Pap q5y (35 - 65) | 4,493 | 0 | 1,573 | 2,921 | 25.88886 | 71.88 | 25.87940 | 24.53011 | 1.31763 | 0.03166 | Dom |
| 26 | VIA q5Y (35-50) | 4,003 | 0 | 1,118 | 2,885 | 25.88885 | 69.38 | 25.87944 | 24.52909 | 1.31895 | 0.03140 | - 13,691.68 |
| 27 | VIA q5Y (30-45) | 4,207 | 0 | 1,342 | 2,865 | 25.88889 | 69.41 | 25.87957 | 24.52854 | 1.31989 | 0.03113 | Dom |
| 28 | Pap q5y (30 - 55) | 4,597 | 0 | 1,765 | 2,831 | 25.88901 | 71.18 | 25.87988 | 24.53144 | 1.31768 | 0.03076 | Dom |
| 29 | VIA q5Y (35-45) + Pap q5y (50 - 60) | 4,209 | 0 | 1,436 | 2,772 | 25.88918 | 71.10 | 25.88018 | 24.53452 | 1.31558 | 0.03008 | Dom |
| 30 | VIA q5Y (35-55) | 4,049 | 0 | 1,286 | 2,763 | 25.88917 | 70.26 | 25.88018 | 24.53435 | 1.31581 | 0.03003 | - 5,799.12 |
| 31 | Pap q5y (30 - 60) | 4,678 | 0 | 1,904 | 2,773 | 25.88918 | 72.02 | 25.88026 | 24.53430 | 1.31589 | 0.03007 | Dom |
| 32 | VIA q5Y (35-50) + Pap q5y (55 - 60) | 4,166 | 0 | 1,430 | 2,736 | 25.88926 | 71.10 | 25.88038 | 24.53587 | 1.31482 | 0.02969 | Dom |
| 33 | VIA q5Y (35-45) + Pap q5y (50 - 65) | 4,289 | 0 | 1,546 | 2,744 | 25.88927 | 71.89 | 25.88038 | 24.53619 | 1.31448 | 0.02971 | Dom |
| 34 | Pap q5y (30 - 65) | 4,758 | 0 | 2,014 | 2,744 | 25.88928 | 72.81 | 25.88046 | 24.53598 | 1.31478 | 0.02970 | Dom |
| 35 | Vac 11Y LT + VIA q5Y (35-45) | 4,131 | 547 | 907 | 2,677 | 25.88927 | 68.50 | 25.88051 | 24.70507 | 1.14630 | 0.02914 | Dom |
| 36 | VIA q5Y (35-50) + Pap q5y (55 - 65) | 4,247 | 0 | 1,539 | 2,708 | 25.88935 | 71.89 | 25.88057 | 24.53752 | 1.31373 | 0.02932 | Dom |
| 37 | VIA q5Y (35-55) + Pap q5y (60 - 65) | 4,218 | 0 | 1,534 | 2,684 | 25.88941 | 71.89 | 25.88070 | 24.53847 | 1.31317 | 0.02906 | Dom |
| 38 | VIA q5Y (30-50) | 4,221 | 0 | 1,548 | 2,673 | 25.88936 | 70.31 | 25.88071 | 24.53615 | 1.31553 | 0.02903 | Dom |
| 39 | VIA q5Y (25-45) | 4,516 | 0 | 1,857 | 2,659 | 25.88938 | 70.34 | 25.88080 | 24.53521 | 1.31682 | 0.02877 | Dom |
| 40 | Pap q5y (25 - 55) | 4,954 | 0 | 2,294 | 2,660 | 25.88942 | 72.11 | 25.88091 | 24.53699 | 1.31511 | 0.02880 | Dom |
| 41 | Vac 11Y LT + Pap q5y (35 - 55) | 4,481 | 547 | 1,319 | 2,614 | 25.88946 | 70.28 | 25.88097 | 24.70870 | 1.14384 | 0.02843 | Dom |
| 42 | Pap q5y (25 - 60) | 5,035 | 0 | 2,433 | 2,602 | 25.88959 | 72.95 | 25.88128 | 24.53984 | 1.31332 | 0.02812 | Dom |
| 43 | Vac 11Y LT + Pap q5y (35 - 60) | 4,569 | 547 | 1,458 | 2,563 | 25.88961 | 71.13 | 25.88130 | 24.71120 | 1.14227 | 0.02783 | Dom |
| 44 | VIA q5Y (20-45) | 5,028 | 0 | 2,468 | 2,561 | 25.88961 | 71.23 | 25.88137 | 24.53833 | 1.31544 | 0.02761 | Dom |
| 45 | VIA q5Y (30-55) | 4,266 | 0 | 1,715 | 2,551 | 25.88968 | 71.18 | 25.88145 | 24.54140 | 1.31239 | 0.02766 | Dom |
| 46 | VIA q5Y (30-45) + Pap q5y (50 - 60) | 4,426 | 0 | 1,866 | 2,560 | 25.88969 | 72.03 | 25.88145 | 24.54158 | 1.31216 | 0.02771 | Dom |
| 47 | Vac 11Y LT + Pap q5y (35 - 65) | 4,653 | 547 | 1,567 | 2,538 | 25.88970 | 71.92 | 25.88148 | 24.71267 | 1.14130 | 0.02750 | Dom |
| 48 | Pap q5y (25 - 65) | 5,115 | 0 | 2,542 | 2,573 | 25.88969 | 73.74 | 25.88148 | 24.54153 | 1.31221 | 0.02774 | Dom |
| 49 | Vac 11Y LT + VIA q5Y (35-50) | 4,168 | 547 | 1,112 | 2,508 | 25.88969 | 69.41 | 25.88151 | 24.71175 | 1.14247 | 0.02729 | Dom |
| 50 | Vac 11Y LT + VIA q5Y (30-45) | 4,375 | 547 | 1,335 | 2,493 | 25.88971 | 69.44 | 25.88161 | 24.71120 | 1.14332 | 0.02708 | Dom |
| 51 | VIA q5Y (30-50) + Pap q5y (55 - 60) | 4,384 | 0 | 1,859 | 2,524 | 25.88977 | 72.03 | 25.88165 | 24.54293 | 1.31140 | 0.02732 | Dom |
| 52 | VIA q5Y (30-45) + Pap q5y (50 - 65) | 4,507 | 0 | 1,975 | 2,532 | 25.88978 | 72.82 | 25.88165 | 24.54325 | 1.31106 | 0.02734 | Dom |
| 53 | Pap q5y (20 - 60) | 5,580 | 0 | 3,059 | 2,521 | 25.88979 | 73.84 | 25.88176 | 24.54244 | 1.31217 | 0.02716 | Dom |
| 54 | VIA q5Y (30-50) + Pap q5y (55 - 65) | 4,465 | 0 | 1,969 | 2,496 | 25.88986 | 72.82 | 25.88184 | 24.54458 | 1.31031 | 0.02696 | Dom |
| 55 | Vac 11Y LT + Pap q5y (30 - 55) | 4,769 | 547 | 1,759 | 2,462 | 25.88983 | 71.22 | 25.88189 | 24.71377 | 1.14138 | 0.02674 | Dom |
| 56 | VIA q5Y (25-50) | 4,530 | 0 | 2,063 | 2,467 | 25.88985 | 71.24 | 25.88194 | 24.54281 | 1.31247 | 0.02666 | Dom |
| 57 | Pap q5y (20 - 65) | 5,661 | 0 | 3,169 | 2,492 | 25.88988 | 74.63 | 25.88196 | 24.54412 | 1.31106 | 0.02678 | Dom |
| 58 | VIA q5Y (30-55) + Pap q5y (60 - 65) | 4,435 | 0 | 1,963 | 2,472 | 25.88991 | 72.82 | 25.88197 | 24.54552 | 1.30976 | 0.02670 | Dom |
| 59 | Vac 11Y LT + VIA q5Y (35-55) | 4,228 | 547 | 1,279 | 2,401 | 25.88996 | 70.29 | 25.88216 | 24.71634 | 1.13972 | 0.02609 | Dom |
| 60 | Vac 11Y LT + VIA q5Y (35-45) + Pap q5y (50 - 60) | 4,387 | 547 | 1,430 | 2,409 | 25.88997 | 71.13 | 25.88216 | 24.71650 | 1.13952 | 0.02614 | Dom |
| 61 | Vac 11Y LT + Pap q5y (30 - 60) | 4,857 | 547 | 1,898 | 2,411 | 25.88998 | 72.06 | 25.88222 | 24.71626 | 1.13981 | 0.02614 | Dom |
| 62 | Vac 11Y LT + VIA q5Y (35-50) + Pap q5y (55 - 60) | 4,348 | 547 | 1,423 | 2,378 | 25.89004 | 71.13 | 25.88233 | 24.71768 | 1.13886 | 0.02580 | Dom |
| 63 | Vac 11Y LT + VIA q5Y (35-45) + Pap q5y (50 - 65) | 4,471 | 547 | 1,539 | 2,384 | 25.89006 | 71.93 | 25.88233 | 24.71796 | 1.13856 | 0.02581 | Dom |
| 64 | Vac 11Y LT + Pap q5y (30 - 65) | 4,941 | 547 | 2,007 | 2,386 | 25.89006 | 72.86 | 25.88239 | 24.71774 | 1.13884 | 0.02582 | Dom |
| 65 | Vac 11Y LT + VIA q5Y (35-50) + Pap q5y (55 - 65) | 4,433 | 547 | 1,533 | 2,353 | 25.89013 | 71.93 | 25.88250 | 24.71912 | 1.13790 | 0.02547 | Dom |
| 66 | VIA q5Y (20-50) | 5,042 | 0 | 2,674 | 2,369 | 25.89008 | 72.13 | 25.88252 | 24.54593 | 1.31108 | 0.02550 | Dom |
| 67 | Vac 11Y LT + VIA q5Y (30-50) | 4,412 | 547 | 1,540 | 2,325 | 25.89013 | 70.34 | 25.88261 | 24.71784 | 1.13952 | 0.02524 | Dom |
| 68 | Vac 11Y LT + VIA q5Y (35-55) + Pap q5y (60 - 65) | 4,406 | 547 | 1,527 | 2,332 | 25.89017 | 71.93 | 25.88261 | 24.71995 | 1.13742 | 0.02525 | Dom |
| 69 | Vac 11Y LT + VIA q5Y (25-45) | 4,711 | 547 | 1,848 | 2,316 | 25.89014 | 70.38 | 25.88267 | 24.71693 | 1.14069 | 0.02505 | Dom |
| 70 | VIA q5Y (25-55) | 4,576 | 0 | 2,231 | 2,345 | 25.89017 | 72.11 | 25.88268 | 24.54806 | 1.30933 | 0.02530 | Dom |
| 71 | VIA q5Y (25-45) + Pap q5y (50 - 60) | 4,736 | 0 | 2,382 | 2,354 | 25.89018 | 72.96 | 25.88268 | 24.54824 | 1.30910 | 0.02535 | Dom |
| 72 | Vac 11Y LT + Pap q5y (25 - 55) | 5,149 | 547 | 2,286 | 2,315 | 25.89018 | 72.15 | 25.88277 | 24.71853 | 1.13917 | 0.02506 | Dom |
| 73 | VIA q5Y (25-45) + Pap q5y (50 - 65) | 4,817 | 0 | 2,491 | 2,325 | 25.89027 | 73.75 | 25.88288 | 24.54991 | 1.30800 | 0.02498 | Dom |
| 74 | VIA q5Y (25-50) + Pap q5y (55 - 65) | 4,774 | 0 | 2,485 | 2,290 | 25.89036 | 73.75 | 25.88307 | 24.55123 | 1.30725 | 0.02459 | Dom |
| 75 | Vac 11Y LT + Pap q5y (25 - 60) | 5,237 | 547 | 2,425 | 2,264 | 25.89033 | 73.00 | 25.88310 | 24.72103 | 1.13761 | 0.02446 | Dom |
| 76 | Vac 11Y LT + VIA q5Y (20-45) | 5,235 | 547 | 2,456 | 2,232 | 25.89033 | 71.28 | 25.88316 | 24.71960 | 1.13950 | 0.02406 | Dom |
| 77 | Vac 11Y LT + Pap q5y (20 - 55) | 5,705 | 547 | 2,912 | 2,245 | 25.89034 | 73.06 | 25.88318 | 24.72075 | 1.13818 | 0.02424 | Dom |
| 78 | VIA q5Y (25-55) + Pap q5y (60 - 65) | 4,745 | 0 | 2,479 | 2,266 | 25.89041 | 73.75 | 25.88320 | 24.55217 | 1.30670 | 0.02433 | Dom |
| 79 | Vac 11Y LT + VIA q5Y (30-55) | 4,473 | 547 | 1,707 | 2,218 | 25.89040 | 71.22 | 25.88325 | 24.72243 | 1.13678 | 0.02405 | Dom |
| 80 | Vac 11Y LT + VIA q5Y (30-45) + Pap q5y (50 - 60) | 4,631 | 547 | 1,858 | 2,226 | 25.89041 | 72.07 | 25.88325 | 24.72259 | 1.13657 | 0.02410 | Dom |
| 81 | VIA q5Y (20-55) | 5,088 | 0 | 2,841 | 2,247 | 25.89040 | 73.01 | 25.88326 | 24.55117 | 1.30794 | 0.02414 | Dom |
| 82 | VIA q5Y (20-45) + Pap q5y (50 - 60) | 5,248 | 0 | 2,992 | 2,256 | 25.89041 | 73.85 | 25.88326 | 24.55135 | 1.30771 | 0.02419 | Dom |
| 83 | Vac 11Y LT + Pap q5y (25 - 65) | 5,321 | 547 | 2,534 | 2,239 | 25.89041 | 73.79 | 25.88327 | 24.72250 | 1.13664 | 0.02413 | Dom |
| 84 | Vac 11Y LT + VIA q5Y (30-50) + Pap q5y (55 - 60) | 4,593 | 547 | 1,851 | 2,195 | 25.89048 | 72.07 | 25.88343 | 24.72376 | 1.13591 | 0.02375 | Dom |
| 85 | Vac 11Y LT + VIA q5Y (30-45) + Pap q5y (50 - 65) | 4,716 | 547 | 1,967 | 2,201 | 25.89049 | 72.86 | 25.88343 | 24.72405 | 1.13561 | 0.02377 | Dom |
| 86 | VIA q5Y (20-50) + Pap q5y (55 - 60) | 5,206 | 0 | 2,986 | 2,220 | 25.89049 | 73.85 | 25.88345 | 24.55270 | 1.30695 | 0.02380 | Dom |
| 87 | VIA q5Y (20-50) + Pap q5y (55 - 60) | 5,206 | 0 | 2,986 | 2,220 | 25.89049 | 73.85 | 25.88345 | 24.55270 | 1.30695 | 0.02380 | Dom |
| 88 | VIA q5Y (20-45) + Pap q5y (50 - 65) | 5,329 | 0 | 3,102 | 2,227 | 25.89050 | 74.64 | 25.88346 | 24.55302 | 1.30661 | 0.02382 | Dom |
| 89 | Vac 11Y LT + Pap q5y (20 - 60) | 5,793 | 547 | 3,051 | 2,195 | 25.89049 | 73.90 | 25.88351 | 24.72325 | 1.13662 | 0.02364 | Dom |
| 90 | Vac 11Y LT + VIA q5Y (30-50) + Pap q5y (55 - 65) | 4,678 | 547 | 1,960 | 2,170 | 25.89056 | 72.87 | 25.88360 | 24.72521 | 1.13496 | 0.02343 | Dom |
| 91 | VIA q5Y (20-50) + Pap q5y (55 - 65) | 5,287 | 0 | 3,095 | 2,191 | 25.89058 | 74.64 | 25.88365 | 24.55435 | 1.30587 | 0.02343 | Dom |
| 92 | Vac 11Y LT + VIA q5Y (25-50) | 4,748 | 547 | 2,053 | 2,148 | 25.89055 | 71.28 | 25.88366 | 24.72356 | 1.13689 | 0.02321 | Dom |
| 93 | Vac 11Y LT + Pap q5y (20 - 65) | 5,877 | 547 | 3,160 | 2,169 | 25.89057 | 74.70 | 25.88368 | 24.72472 | 1.13565 | 0.02331 | Dom |
| 94 | Vac 11Y LT + VIA q5Y (30-55) + Pap q5y (60 - 65) | 4,651 | 547 | 1,955 | 2,149 | 25.89061 | 72.87 | 25.88371 | 24.72603 | 1.13447 | 0.02321 | Dom |
| 95 | VIA q5Y (20-55) + Pap q5y (60 - 65) | 5,257 | 0 | 3,090 | 2,168 | 25.89064 | 74.64 | 25.88378 | 24.55529 | 1.30531 | 0.02317 | Dom |
| 96 | Vac 11Y LT + VIA q5Y (20-50) | 5,272 | 547 | 2,661 | 2,064 | 25.89075 | 72.18 | 25.88415 | 24.72623 | 1.13570 | 0.02222 | Dom |
| 97 | Vac 11Y LT + VIA q5Y (25-55) | 4,809 | 547 | 2,220 | 2,041 | 25.89083 | 72.16 | 25.88431 | 24.72815 | 1.13415 | 0.02202 | Dom |
| 98 | Vac 11Y LT + VIA q5Y (25-45) + Pap q5y (50 - 60) | 4,968 | 547 | 2,371 | 2,049 | 25.89084 | 73.01 | 25.88431 | 24.72831 | 1.13394 | 0.02206 | Dom |
| 99 | Vac 11Y LT + VIA q5Y (25-45) + Pap q5y (50 - 65) | 5,052 | 547 | 2,480 | 2,024 | 25.89092 | 73.80 | 25.88448 | 24.72977 | 1.13298 | 0.02174 | Dom |
| 100 | Vac 11Y LT + VIA q5Y (25-50) + Pap q5y (55 - 65) | 5,014 | 547 | 2,474 | 1,993 | 25.89099 | 73.80 | 25.88465 | 24.73093 | 1.13233 | 0.02140 | Dom |
| 101 | Vac 11Y LT + VIA q5Y (25-55) + Pap q5y (60 - 65) | 4,987 | 547 | 2,468 | 1,972 | 25.89104 | 73.80 | 25.88476 | 24.73175 | 1.13184 | 0.02117 | Dom |
| 102 | Vac 11Y LT + VIA q5Y (20-55) | 5,333 | 547 | 2,828 | 1,957 | 25.89102 | 73.07 | 25.88480 | 24.73081 | 1.13296 | 0.02103 | Dom |
| 103 | Vac 11Y LT + VIA q5Y (20-45) + Pap q5y (50 - 60) | 5,492 | 547 | 2,979 | 1,965 | 25.89103 | 73.91 | 25.88480 | 24.73097 | 1.13276 | 0.02107 | Dom |
| 104 | Vac 11Y LT + VIA q5Y (20-50) + Pap q5y (55 - 60) | 5,453 | 547 | 2,972 | 1,934 | 25.89110 | 73.91 | 25.88497 | 24.73215 | 1.13210 | 0.02073 | Dom |
| 105 | Vac 11Y LT + VIA q5Y (20-50) + Pap q5y (55 - 60) | 5,453 | 547 | 2,972 | 1,934 | 25.89110 | 73.91 | 25.88497 | 24.73215 | 1.13210 | 0.02073 | Dom |
| 106 | Vac 11Y LT + VIA q5Y (20-45) + Pap q5y (50 - 65) | 5,576 | 547 | 3,088 | 1,940 | 25.89111 | 74.71 | 25.88498 | 24.73243 | 1.13180 | 0.02075 | Dom |
| 107 | Vac 11Y LT + VIA q5Y (20-50) + Pap q5y (55 - 65) | 5,538 | 547 | 3,082 | 1,909 | 25.89118 | 74.71 | 25.88515 | 24.73359 | 1.13114 | 0.02041 | Dom |
| 108 | Vac 11Y LT + VIA q5Y (20-55) + Pap q5y (60 - 65) | 5,511 | 547 | 3,076 | 1,888 | 25.89123 | 74.71 | 25.88526 | 24.73441 | 1.13066 | 0.02018 | Dom |

Table 2-C. Cost and health outcomes of competing strategies at 8% screening and 80% vaccination coverages

|  | Strategies | Cost | | | | LY s | LE | QALYs | | | | ICER |
| --- | --- | --- | --- | --- | --- | --- | --- | --- | --- | --- | --- | --- |
|  |  | Total | prevention | | treatment of  cervical cancer |  |  | Total | Healthy | HPV infection - CIN | cervical cancer |  |
|  |  |  | vaccination | CIN |  |  |  |  |  |  |  |  |
| 1 | Pap q5y (20 - 55) | 4,087 | 0 | 0 | 4,087 | 25.88600 | 65.71 | 25.87236 | 24.48558 | 1.34230 | 0.04448 | Dom |
| 2 | Vac 25Y boost q10 | 5,324 | 1,988 | 0 | 3,336 | 25.88764 | 65.73 | 25.87645 | 24.66069 | 1.17930 | 0.03646 | Dom |
| 3 | Vac 25Y boost q15 | 5,065 | 1,729 | 0 | 3,336 | 25.88764 | 65.73 | 25.87645 | 24.66069 | 1.17930 | 0.03646 | Dom |
| 4 | Vac 25Y boost q20 | 4,917 | 1,581 | 0 | 3,336 | 25.88764 | 65.73 | 25.87645 | 24.66069 | 1.17930 | 0.03646 | Dom |
| 5 | Vac 25Y LT | 4,648 | 1,313 | 0 | 3,335 | 25.88764 | 65.73 | 25.87645 | 24.66069 | 1.17930 | 0.03646 | Dom |
| 6 | VIA q5Y (35-45) | 3,991 | 0 | 912 | 3,079 | 25.88838 | 68.48 | 25.87829 | 24.52144 | 1.32334 | 0.03352 | Dom |
| 7 | Vac 20Y boost q10 | 5,268 | 2,299 | 0 | 2,970 | 25.88849 | 65.73 | 25.87852 | 24.79439 | 1.05164 | 0.03249 | Dom |
| 8 | Vac 20Y boost q15 | 4,938 | 1,969 | 0 | 2,970 | 25.88849 | 65.73 | 25.87852 | 24.79439 | 1.05164 | 0.03249 | Dom |
| 9 | Vac 20Y boost q20 | 4,777 | 1,807 | 0 | 2,969 | 25.88849 | 65.73 | 25.87852 | 24.79439 | 1.05164 | 0.03249 | Dom |
| 10 | Vac 20Y LT | 4,484 | 1,515 | 0 | 2,969 | 25.88849 | 65.73 | 25.87852 | 24.79439 | 1.05164 | 0.03249 | Dom |
| 11 | Pap q5y (35 - 55) | 4,332 | 0 | 1,324 | 3,008 | 25.88859 | 70.25 | 25.87882 | 24.52556 | 1.32054 | 0.03272 | Reference |
| 12 | Pap q5y (35 - 60) | 4,413 | 0 | 1,463 | 2,950 | 25.88876 | 71.09 | 25.87920 | 24.52842 | 1.31874 | 0.03203 | Dom |
| 13 | Pap q5y (35 - 65) | 4,493 | 0 | 1,573 | 2,921 | 25.88886 | 71.88 | 25.87940 | 24.53011 | 1.31763 | 0.03166 | Dom |
| 14 | VIA q5Y (35-50) | 4,003 | 0 | 1,118 | 2,885 | 25.88885 | 69.38 | 25.87944 | 24.52909 | 1.31895 | 0.03140 | Dom |
| 15 | VIA q5Y (30-45) | 4,207 | 0 | 1,342 | 2,865 | 25.88889 | 69.41 | 25.87957 | 24.52854 | 1.31989 | 0.03113 | Dom |
| 16 | Pap q5y (30 - 55) | 4,597 | 0 | 1,765 | 2,831 | 25.88901 | 71.18 | 25.87988 | 24.53144 | 1.31768 | 0.03076 | Dom |
| 17 | VIA q5Y (35-45) + Pap q5y (50 - 60) | 4,209 | 0 | 1,436 | 2,772 | 25.88918 | 71.10 | 25.88018 | 24.53452 | 1.31558 | 0.03008 | Dom |
| 18 | VIA q5Y (35-55) | 4,048 | 0 | 129 | 3,918 | 25.88641 | 70.22 | 25.87337 | 24.49188 | 1.338851 | 0.042638 | -191,099.19 |
| 19 | Pap q5y (30 - 60) | 4,678 | 0 | 1,904 | 2,773 | 25.88918 | 72.02 | 25.88026 | 24.53430 | 1.31589 | 0.03007 | Dom |
| 20 | VIA q5Y (35-50) + Pap q5y (55 - 60) | 4,166 | 0 | 1,430 | 2,736 | 25.88926 | 71.10 | 25.88038 | 24.53587 | 1.31482 | 0.02969 | Dom |
| 21 | VIA q5Y (35-45) + Pap q5y (50 - 65) | 4,289 | 0 | 1,546 | 2,744 | 25.88927 | 71.89 | 25.88038 | 24.53619 | 1.31448 | 0.02971 | Dom |
| 22 | Pap q5y (30 - 65) | 4,758 | 0 | 2,014 | 2,744 | 25.88928 | 72.81 | 25.88046 | 24.53598 | 1.31478 | 0.02970 | Dom |
| 23 | VIA q5Y (35-50) + Pap q5y (55 - 65) | 4,247 | 0 | 1,539 | 2,708 | 25.88935 | 71.89 | 25.88057 | 24.53752 | 1.31373 | 0.02932 | Dom |
| 24 | VIA q5Y (35-55) + Pap q5y (60 - 65) | 4,218 | 0 | 1,534 | 2,684 | 25.88941 | 71.89 | 25.88070 | 24.53847 | 1.31317 | 0.02906 | Dom |
| 25 | VIA q5Y (30-50) | 4,221 | 0 | 1,548 | 2,673 | 25.88936 | 70.31 | 25.88071 | 24.53615 | 1.31553 | 0.02903 | Dom |
| 26 | VIA q5Y (25-45) | 4,516 | 0 | 1,857 | 2,659 | 25.88938 | 70.34 | 25.88080 | 24.53521 | 1.31682 | 0.02877 | Dom |
| 27 | Pap q5y (25 - 55) | 4,954 | 0 | 2,294 | 2,660 | 25.88942 | 72.11 | 25.88091 | 24.53699 | 1.31511 | 0.02880 | Dom |
| 28 | Pap q5y (25 - 60) | 5,035 | 0 | 2,433 | 2,602 | 25.88959 | 72.95 | 25.88128 | 24.53984 | 1.31332 | 0.02812 | Dom |
| 29 | VIA q5Y (20-45) | 5,028 | 0 | 2,468 | 2,561 | 25.88961 | 71.23 | 25.88137 | 24.53833 | 1.31544 | 0.02761 | Dom |
| 30 | VIA q5Y (30-55) | 4,266 | 0 | 1,715 | 2,551 | 25.88968 | 71.18 | 25.88145 | 24.54140 | 1.31239 | 0.02766 | Dom |
| 31 | VIA q5Y (30-45) + Pap q5y (50 - 60) | 4,426 | 0 | 1,866 | 2,560 | 25.88969 | 72.03 | 25.88145 | 24.54158 | 1.31216 | 0.02771 | Dom |
| 32 | Pap q5y (25 - 65) | 5,115 | 0 | 2,542 | 2,573 | 25.88969 | 73.74 | 25.88148 | 24.54153 | 1.31221 | 0.02774 | Dom |
| 33 | VIA q5Y (30-50) + Pap q5y (55 - 60) | 4,384 | 0 | 1,859 | 2,524 | 25.88977 | 72.03 | 25.88165 | 24.54293 | 1.31140 | 0.02732 | Dom |
| 34 | VIA q5Y (30-45) + Pap q5y (50 - 65) | 4,507 | 0 | 1,975 | 2,532 | 25.88978 | 72.82 | 25.88165 | 24.54325 | 1.31106 | 0.02734 | Dom |
| 35 | Pap q5y (20 - 60) | 5,580 | 0 | 3,059 | 2,521 | 25.88979 | 73.84 | 25.88176 | 24.54244 | 1.31217 | 0.02716 | Dom |
| 36 | VIA q5Y (30-50) + Pap q5y (55 - 65) | 4,465 | 0 | 1,969 | 2,496 | 25.88986 | 72.82 | 25.88184 | 24.54458 | 1.31031 | 0.02696 | Dom |
| 37 | VIA q5Y (25-50) | 4,530 | 0 | 2,063 | 2,467 | 25.88985 | 71.24 | 25.88194 | 24.54281 | 1.31247 | 0.02666 | Dom |
| 38 | Pap q5y (20 - 65) | 5,661 | 0 | 3,169 | 2,492 | 25.88988 | 74.63 | 25.88196 | 24.54412 | 1.31106 | 0.02678 | Dom |
| 39 | VIA q5Y (30-55) + Pap q5y (60 - 65) | 4,435 | 0 | 1,963 | 2,472 | 25.88991 | 72.82 | 25.88197 | 24.54552 | 1.30976 | 0.02670 | Dom |
| 40 | VIA q5Y (20-50) | 5,042 | 0 | 2,674 | 2,369 | 25.89008 | 72.13 | 25.88252 | 24.54593 | 1.31108 | 0.02550 | Dom |
| 41 | VIA q5Y (25-55) | 4,576 | 0 | 2,231 | 2,345 | 25.89017 | 72.11 | 25.88268 | 24.54806 | 1.30933 | 0.02530 | Dom |
| 42 | VIA q5Y (25-45) + Pap q5y (50 - 60) | 4,736 | 0 | 2,382 | 2,354 | 25.89018 | 72.96 | 25.88268 | 24.54824 | 1.30910 | 0.02535 | Dom |
| 43 | VIA q5Y (25-45) + Pap q5y (50 - 65) | 4,817 | 0 | 2,491 | 2,325 | 25.89027 | 73.75 | 25.88288 | 24.54991 | 1.30800 | 0.02498 | Dom |
| 44 | VIA q5Y (25-50) + Pap q5y (55 - 65) | 4,774 | 0 | 2,485 | 2,290 | 25.89036 | 73.75 | 25.88307 | 24.55123 | 1.30725 | 0.02459 | Dom |
| 45 | VIA q5Y (25-55) + Pap q5y (60 - 65) | 4,745 | 0 | 2,479 | 2,266 | 25.89041 | 73.75 | 25.88320 | 24.55217 | 1.30670 | 0.02433 | Dom |
| 46 | VIA q5Y (20-55) | 5,088 | 0 | 2,841 | 2,247 | 25.89040 | 73.01 | 25.88326 | 24.55117 | 1.30794 | 0.02414 | Dom |
| 47 | VIA q5Y (20-45) + Pap q5y (50 - 60) | 5,248 | 0 | 2,992 | 2,256 | 25.89041 | 73.85 | 25.88326 | 24.55135 | 1.30771 | 0.02419 | Dom |
| 48 | VIA q5Y (20-50) + Pap q5y (55 - 60) | 5,206 | 0 | 2,986 | 2,220 | 25.89049 | 73.85 | 25.88345 | 24.55270 | 1.30695 | 0.02380 | Dom |
| 49 | VIA q5Y (20-50) + Pap q5y (55 - 60) | 5,206 | 0 | 2,986 | 2,220 | 25.89049 | 73.85 | 25.88345 | 24.55270 | 1.30695 | 0.02380 | Dom |
| 50 | VIA q5Y (20-45) + Pap q5y (50 - 65) | 5,329 | 0 | 3,102 | 2,227 | 25.89050 | 74.64 | 25.88346 | 24.55302 | 1.30661 | 0.02382 | Dom |
| 51 | Vac 13Y boost q10 | 5,240 | 3,145 | 0 | 2,095 | 25.89050 | 65.75 | 25.88351 | 25.17302 | 0.68767 | 0.02283 | Dom |
| 52 | Vac 13Y boost q15 | 4,762 | 2,668 | 0 | 2,095 | 25.89050 | 65.75 | 25.88351 | 25.17302 | 0.68767 | 0.02283 | Dom |
| 53 | Vac 13Y boost q20 | 4,531 | 2,436 | 0 | 2,095 | 25.89050 | 65.75 | 25.88351 | 25.17302 | 0.68767 | 0.02283 | Dom |
| 54 | Vac 13Y LT | 4,114 | 2,020 | 0 | 2,094 | 25.89050 | 65.75 | 25.88351 | 25.17302 | 0.68767 | 0.02283 | 2,407.37 |
| 55 | VIA q5Y (20-50) + Pap q5y (55 - 65) | 5,287 | 0 | 3,095 | 2,191 | 25.89058 | 74.64 | 25.88365 | 24.55435 | 1.30587 | 0.02343 | Dom |
| 56 | VIA q5Y (20-55) + Pap q5y (60 - 65) | 5,257 | 0 | 3,090 | 2,168 | 25.89064 | 74.64 | 25.88378 | 24.55529 | 1.30531 | 0.02317 | Dom |
| 57 | Vac 12Y boost q10 | 5,328 | 3,303 | 0 | 2,025 | 25.89066 | 65.75 | 25.88391 | 25.20500 | 0.65686 | 0.02205 | Dom |
| 58 | Vac 12Y boost q15 | 4,823 | 2,798 | 0 | 2,025 | 25.89066 | 65.75 | 25.88391 | 25.20500 | 0.65686 | 0.02205 | Dom |
| 59 | Vac 12Y boost q20 | 4,578 | 2,553 | 0 | 2,025 | 25.89066 | 65.75 | 25.88391 | 25.20500 | 0.65686 | 0.02205 | Dom |
| 60 | Vac 12Y LT | 4,139 | 2,114 | 0 | 2,025 | 25.89066 | 65.75 | 25.88391 | 25.20500 | 0.65686 | 0.02205 | Dom |
| 61 | Vac 11Y boost q10 | 5,400 | 3,447 | 0 | 1,953 | 25.89083 | 65.75 | 25.88433 | 25.23885 | 0.62424 | 0.02123 | Dom |
| 62 | Vac 11Y boost q15 | 4,863 | 2,910 | 0 | 1,953 | 25.89083 | 65.75 | 25.88433 | 25.23885 | 0.62424 | 0.02123 | Dom |
| 63 | Vac 11Y boost q20 | 4,604 | 2,651 | 0 | 1,953 | 25.89083 | 65.75 | 25.88433 | 25.23885 | 0.62424 | 0.02123 | Dom |
| 64 | Vac 11Y LT | 4,141 | 2,189 | 0 | 1,953 | 25.89083 | 65.75 | 25.88433 | 25.23885 | 0.62424 | 0.02123 | 4,658.65 |
| 65 | Vac 11Y LT + VIA q5Y (35-45) | 4,553 | 2,189 | 893 | 1,472 | 25.89196 | 68.58 | 25.88716 | 25.25597 | 0.61518 | 0.01600 | Dom |
| 66 | Vac 11Y LT + Pap q5y (35 - 55) | 4,928 | 2,189 | 1,305 | 1,434 | 25.89207 | 70.38 | 25.88743 | 25.25810 | 0.61376 | 0.01558 | Dom |
| 67 | Vac 11Y LT + Pap q5y (35 - 60) | 5,037 | 2,189 | 1,443 | 1,405 | 25.89216 | 71.23 | 25.88762 | 25.25952 | 0.61286 | 0.01523 | Dom |
| 68 | Vac 11Y LT + Pap q5y (35 - 65) | 5,131 | 2,189 | 1,552 | 1,390 | 25.89221 | 72.04 | 25.88772 | 25.26037 | 0.61231 | 0.01505 | Dom |
| 69 | Vac 11Y LT + VIA q5Y (35-50) | 4,660 | 2,189 | 1,095 | 1,377 | 25.89219 | 69.49 | 25.88772 | 25.25973 | 0.61303 | 0.01496 | Dom |
| 70 | Vac 11Y LT + VIA q5Y (30-45) | 4,879 | 2,189 | 1,315 | 1,376 | 25.89219 | 69.53 | 25.88773 | 25.25917 | 0.61363 | 0.01493 | Dom |
| 71 | Vac 11Y LT + Pap q5y (30 - 55) | 5,285 | 2,189 | 1,742 | 1,355 | 25.89226 | 71.33 | 25.88791 | 25.26075 | 0.61247 | 0.01469 | Dom |
| 72 | Vac 11Y LT + VIA q5Y (35-55) | 4,765 | 2,189 | 1,260 | 1,316 | 25.89235 | 70.38 | 25.88809 | 25.26234 | 0.61147 | 0.01428 | Dom |
| 73 | Vac 11Y LT + VIA q5Y (35-45) + Pap q5y (50 - 60) | 4,920 | 2,189 | 1,411 | 1,320 | 25.89236 | 71.24 | 25.88809 | 25.26243 | 0.61135 | 0.01430 | Dom |
| 74 | Vac 11Y LT + Pap q5y (30 - 60) | 5,394 | 2,189 | 1,880 | 1,326 | 25.89235 | 72.19 | 25.88810 | 25.26217 | 0.61158 | 0.01435 | Dom |
| 75 | Vac 11Y LT + VIA q5Y (35-50) + Pap q5y (55 - 60) | 4,895 | 2,189 | 1,404 | 1,302 | 25.89240 | 71.24 | 25.88819 | 25.26310 | 0.61098 | 0.01411 | Dom |
| 76 | Vac 11Y LT + VIA q5Y (35-45) + Pap q5y (50 - 65) | 5,015 | 2,189 | 1,520 | 1,306 | 25.89240 | 72.04 | 25.88819 | 25.26327 | 0.61080 | 0.01412 | Dom |
| 77 | Vac 11Y LT + Pap q5y (30 - 65) | 5,489 | 2,189 | 1,989 | 1,311 | 25.89240 | 72.99 | 25.88820 | 25.26301 | 0.61102 | 0.01417 | Dom |
| 78 | Vac 11Y LT + VIA q5Y (25-45) | 5,295 | 2,189 | 1,821 | 1,285 | 25.89241 | 70.49 | 25.88827 | 25.26208 | 0.61229 | 0.01389 | Dom |
| 79 | Vac 11Y LT + VIA q5Y (35-50) + Pap q5y (55 - 65) | 4,989 | 2,189 | 1,512 | 1,288 | 25.89244 | 72.04 | 25.88829 | 25.26392 | 0.61043 | 0.01393 | Dom |
| 80 | Vac 11Y LT + VIA q5Y (30-50) | 4,987 | 2,189 | 1,517 | 1,281 | 25.89242 | 70.45 | 25.88829 | 25.26291 | 0.61149 | 0.01389 | Dom |
| 81 | Vac 11Y LT + VIA q5Y (35-55) + Pap q5y (60 - 65) | 4,971 | 2,189 | 1,506 | 1,276 | 25.89247 | 72.04 | 25.88835 | 25.26439 | 0.61016 | 0.01380 | Dom |
| 82 | Vac 11Y LT + Pap q5y (25 - 55) | 5,734 | 2,189 | 2,265 | 1,280 | 25.89244 | 72.30 | 25.88836 | 25.26317 | 0.61135 | 0.01384 | Dom |
| 83 | Vac 11Y LT + VIA q5Y (20-45) | 5,855 | 2,189 | 2,422 | 1,244 | 25.89250 | 71.44 | 25.88851 | 25.26339 | 0.61171 | 0.01341 | Dom |
| 84 | Vac 11Y LT + Pap q5y (25 - 60) | 5,842 | 2,189 | 2,403 | 1,251 | 25.89253 | 73.15 | 25.88855 | 25.26459 | 0.61046 | 0.01350 | Dom |
| 85 | Vac 11Y LT + Pap q5y (20 - 55) | 6,322 | 2,189 | 2,887 | 1,246 | 25.89252 | 73.24 | 25.88856 | 25.26426 | 0.61086 | 0.01344 | Dom |
| 86 | Vac 11Y LT + Pap q5y (25 - 65) | 5,937 | 2,189 | 2,511 | 1,236 | 25.89258 | 73.95 | 25.88865 | 25.26543 | 0.60990 | 0.01331 | Dom |
| 87 | Vac 11Y LT + VIA q5Y (30-55) | 5,091 | 2,189 | 1,682 | 1,221 | 25.89258 | 71.34 | 25.88866 | 25.26551 | 0.60993 | 0.01321 | Dom |
| 88 | Vac 11Y LT + VIA q5Y (30-45) + Pap q5y (50 - 60) | 5,247 | 2,189 | 1,833 | 1,225 | 25.89259 | 72.19 | 25.88866 | 25.26561 | 0.60981 | 0.01324 | Dom |
| 89 | Vac 11Y LT + Pap q5y (20 - 60) | 6,431 | 2,189 | 3,025 | 1,217 | 25.89261 | 74.10 | 25.88875 | 25.26568 | 0.60997 | 0.01309 | Dom |
| 90 | Vac 11Y LT + VIA q5Y (30-50) + Pap q5y (55 - 60) | 5,222 | 2,189 | 1,826 | 1,207 | 25.89263 | 72.19 | 25.88876 | 25.26627 | 0.60944 | 0.01305 | Dom |
| 91 | Vac 11Y LT + VIA q5Y (30-45) + Pap q5y (50 - 65) | 5,341 | 2,189 | 1,942 | 1,211 | 25.89263 | 73.00 | 25.88876 | 25.26645 | 0.60926 | 0.01305 | Dom |
| 92 | Vac 11Y LT + VIA q5Y (25-50) | 5,403 | 2,189 | 2,023 | 1,191 | 25.89264 | 71.41 | 25.88883 | 25.26582 | 0.61015 | 0.01286 | Dom |
| 93 | Vac 11Y LT + Pap q5y (20 - 65) | 6,525 | 2,189 | 3,134 | 1,202 | 25.89265 | 74.90 | 25.88885 | 25.26652 | 0.60942 | 0.01291 | Dom |
| 94 | Vac 11Y LT + VIA q5Y (30-50) + Pap q5y (55 - 65) | 5,316 | 2,189 | 1,935 | 1,193 | 25.89267 | 73.00 | 25.88886 | 25.26710 | 0.60890 | 0.01286 | Dom |
| 95 | Vac 11Y LT + VIA q5Y (30-55) + Pap q5y (60 - 65) | 5,298 | 2,189 | 1,928 | 1,181 | 25.89270 | 73.00 | 25.88892 | 25.26757 | 0.60862 | 0.01273 | Dom |
| 96 | Vac 11Y LT + VIA q5Y (20-50) | 5,962 | 2,189 | 2,624 | 1,150 | 25.89274 | 72.36 | 25.88907 | 25.26713 | 0.60957 | 0.01237 | Dom |
| 97 | Vac 11Y LT + VIA q5Y (25-55) | 5,507 | 2,189 | 2,188 | 1,131 | 25.89280 | 72.30 | 25.88920 | 25.26842 | 0.60860 | 0.01218 | Dom |
| 98 | Vac 11Y LT + VIA q5Y (25-45) + Pap q5y (50 - 60) | 5,663 | 2,189 | 2,339 | 1,135 | 25.89280 | 73.15 | 25.88920 | 25.26852 | 0.60848 | 0.01220 | Dom |
| 99 | Vac 11Y LT + VIA q5Y (25-45) + Pap q5y (50 - 65) | 5,757 | 2,189 | 2,448 | 1,121 | 25.89285 | 73.96 | 25.88930 | 25.26935 | 0.60793 | 0.01202 | Dom |
| 100 | Vac 11Y LT + VIA q5Y (25-50) + Pap q5y (55 - 65) | 5,732 | 2,189 | 2,440 | 1,103 | 25.89289 | 73.96 | 25.88939 | 25.27001 | 0.60756 | 0.01183 | Dom |
| 101 | Vac 11Y LT + VIA q5Y (20-55) | 6,067 | 2,189 | 2,789 | 1,089 | 25.89289 | 73.25 | 25.88944 | 25.26973 | 0.60802 | 0.01169 | Dom |
| 102 | Vac 11Y LT + VIA q5Y (20-45) + Pap q5y (50 - 60) | 6,222 | 2,189 | 2,940 | 1,094 | 25.89290 | 74.10 | 25.88944 | 25.26983 | 0.60789 | 0.01172 | Dom |
| 103 | Vac 11Y LT + VIA q5Y (25-55) + Pap q5y (60 - 65) | 5,714 | 2,189 | 2,434 | 1,091 | 25.89291 | 73.96 | 25.88946 | 25.27047 | 0.60728 | 0.01170 | Dom |
| 104 | Vac 11Y LT + VIA q5Y (20-50) + Pap q5y (55 - 60) | 6,197 | 2,189 | 2,933 | 1,076 | 25.89294 | 74.10 | 25.88954 | 25.27049 | 0.60752 | 0.01153 | Dom |
| 105 | Vac 11Y LT + VIA q5Y (20-50) + Pap q5y (55 - 60) | 6,197 | 2,189 | 2,933 | 1,076 | 25.89294 | 74.10 | 25.88954 | 25.27049 | 0.60752 | 0.01153 | Dom |
| 106 | Vac 11Y LT + VIA q5Y (20-45) + Pap q5y (50 - 65) | 6,317 | 2,189 | 3,049 | 1,079 | 25.89294 | 74.91 | 25.88954 | 25.27066 | 0.60734 | 0.01153 | Dom |
| 107 | Vac 11Y LT + VIA q5Y (20-50) + Pap q5y (55 - 65) | 6,292 | 2,189 | 3,041 | 1,062 | 25.89298 | 74.91 | 25.88963 | 25.27131 | 0.60698 | 0.01134 | Dom |
| 108 | Vac 11Y LT + VIA q5Y (20-55) + Pap q5y (60 - 65) | 6,274 | 2,189 | 3,035 | 1,050 | 25.89301 | 74.91 | 25.88970 | 25.27178 | 0.60670 | 0.01121 | Dom |

Table 2-D. Cost and health outcomes of competing strategies at 80% screening and 80% vaccination coverages

|  | Strategies | Cost | | | | LY s | LE | QALYs | | | | ICER |
| --- | --- | --- | --- | --- | --- | --- | --- | --- | --- | --- | --- | --- |
|  |  | Total | prevention | | treatment of  cervical cancer |  |  | Total | Healthy | HPV infection - CIN | cervical cancer |  |
|  |  |  | vaccination | CIN |  |  |  |  |  |  |  |  |
| 1 | Pap q5y (20 - 55) | 4,087 | 0 | 0 | 4,087 | 25.88600 | 65.71 | 25.87236 | 24.48558 | 1.34230 | 0.04448 | Dom |
| 2 | Vac 25Y boost q10 | 5,324 | 1,988 | 0 | 3,336 | 25.88764 | 65.73 | 25.87645 | 24.66069 | 1.17930 | 0.03646 | Dom |
| 3 | Vac 25Y boost q15 | 5,065 | 1,729 | 0 | 3,336 | 25.88764 | 65.73 | 25.87645 | 24.66069 | 1.17930 | 0.03646 | Dom |
| 4 | Vac 25Y boost q20 | 4,917 | 1,581 | 0 | 3,336 | 25.88764 | 65.73 | 25.87645 | 24.66069 | 1.17930 | 0.03646 | Dom |
| 5 | Vac 25Y LT | 4,648 | 1,313 | 0 | 3,335 | 25.88764 | 65.73 | 25.87645 | 24.66069 | 1.17930 | 0.03646 | Dom |
| 6 | VIA q5Y (35-45) | 3,991 | 0 | 912 | 3,079 | 25.88838 | 68.48 | 25.87829 | 24.52144 | 1.32334 | 0.03352 | -19,189.52 |
| 7 | Vac 20Y boost q10 | 5,268 | 2,299 | 0 | 2,970 | 25.88849 | 65.73 | 25.87852 | 24.79439 | 1.05164 | 0.03249 | Dom |
| 8 | Vac 20Y boost q15 | 4,938 | 1,969 | 0 | 2,970 | 25.88849 | 65.73 | 25.87852 | 24.79439 | 1.05164 | 0.03249 | Dom |
| 9 | Vac 20Y boost q20 | 4,777 | 1,807 | 0 | 2,969 | 25.88849 | 65.73 | 25.87852 | 24.79439 | 1.05164 | 0.03249 | Dom |
| 10 | Vac 20Y LT | 4,484 | 1,515 | 0 | 2,969 | 25.88849 | 65.73 | 25.87852 | 24.79439 | 1.05164 | 0.03249 | Dom |
| 11 | Pap q5y (35 - 55) | 4,332 | 0 | 1,324 | 3,008 | 25.88859 | 70.25 | 25.87882 | 24.52556 | 1.32054 | 0.03272 | Reference |
| 12 | Pap q5y (35 - 60) | 4,413 | 0 | 1,463 | 2,950 | 25.88876 | 71.09 | 25.87920 | 24.52842 | 1.31874 | 0.03203 | Dom |
| 13 | Pap q5y (35 - 65) | 4,493 | 0 | 1,573 | 2,921 | 25.88886 | 71.88 | 25.87940 | 24.53011 | 1.31763 | 0.03166 | Dom |
| 14 | VIA q5Y (35-50) | 4,003 | 0 | 1,118 | 2,885 | 25.88885 | 69.38 | 25.87944 | 24.52909 | 1.31895 | 0.03140 | - 13,691.68 |
| 15 | VIA q5Y (30-45) | 4,207 | 0 | 1,342 | 2,865 | 25.88889 | 69.41 | 25.87957 | 24.52854 | 1.31989 | 0.03113 | Dom |
| 16 | Pap q5y (30 - 55) | 4,597 | 0 | 1,765 | 2,831 | 25.88901 | 71.18 | 25.87988 | 24.53144 | 1.31768 | 0.03076 | Dom |
| 17 | VIA q5Y (35-45) + Pap q5y (50 - 60) | 4,209 | 0 | 1,436 | 2,772 | 25.88918 | 71.10 | 25.88018 | 24.53452 | 1.31558 | 0.03008 | Dom |
| 18 | VIA q5Y (35-55) | 4,049 | 0 | 1,286 | 2,763 | 25.88917 | 70.26 | 25.88018 | 24.53435 | 1.31581 | 0.03003 | Dom |
| 19 | Pap q5y (30 - 60) | 4,678 | 0 | 1,904 | 2,773 | 25.88918 | 72.02 | 25.88026 | 24.53430 | 1.31589 | 0.03007 | Dom |
| 20 | VIA q5Y (35-50) + Pap q5y (55 - 60) | 4,166 | 0 | 1,430 | 2,736 | 25.88926 | 71.10 | 25.88038 | 24.53587 | 1.31482 | 0.02969 | Dom |
| 21 | VIA q5Y (35-45) + Pap q5y (50 - 65) | 4,289 | 0 | 1,546 | 2,744 | 25.88927 | 71.89 | 25.88038 | 24.53619 | 1.31448 | 0.02971 | Dom |
| 22 | Pap q5y (30 - 65) | 4,758 | 0 | 2,014 | 2,744 | 25.88928 | 72.81 | 25.88046 | 24.53598 | 1.31478 | 0.02970 | Dom |
| 23 | VIA q5Y (35-50) + Pap q5y (55 - 65) | 4,247 | 0 | 1,539 | 2,708 | 25.88935 | 71.89 | 25.88057 | 24.53752 | 1.31373 | 0.02932 | Dom |
| 24 | VIA q5Y (35-55) + Pap q5y (60 - 65) | 4,218 | 0 | 1,534 | 2,684 | 25.88941 | 71.89 | 25.88070 | 24.53847 | 1.31317 | 0.02906 | Dom |
| 25 | VIA q5Y (30-50) | 4,221 | 0 | 1,548 | 2,673 | 25.88936 | 70.31 | 25.88071 | 24.53615 | 1.31553 | 0.02903 | Dom |
| 26 | VIA q5Y (25-45) | 4,516 | 0 | 1,857 | 2,659 | 25.88938 | 70.34 | 25.88080 | 24.53521 | 1.31682 | 0.02877 | Dom |
| 27 | Pap q5y (25 - 55) | 4,954 | 0 | 2,294 | 2,660 | 25.88942 | 72.11 | 25.88091 | 24.53699 | 1.31511 | 0.02880 | Dom |
| 28 | Pap q5y (25 - 60) | 5,035 | 0 | 2,433 | 2,602 | 25.88959 | 72.95 | 25.88128 | 24.53984 | 1.31332 | 0.02812 | Dom |
| 29 | VIA q5Y (20-45) | 5,028 | 0 | 2,468 | 2,561 | 25.88961 | 71.23 | 25.88137 | 24.53833 | 1.31544 | 0.02761 | Dom |
| 30 | VIA q5Y (30-55) | 4,266 | 0 | 1,715 | 2,551 | 25.88968 | 71.18 | 25.88145 | 24.54140 | 1.31239 | 0.02766 | Dom |
| 31 | VIA q5Y (30-45) + Pap q5y (50 - 60) | 4,426 | 0 | 1,866 | 2,560 | 25.88969 | 72.03 | 25.88145 | 24.54158 | 1.31216 | 0.02771 | Dom |
| 32 | Pap q5y (25 - 65) | 5,115 | 0 | 2,542 | 2,573 | 25.88969 | 73.74 | 25.88148 | 24.54153 | 1.31221 | 0.02774 | Dom |
| 33 | VIA q5Y (30-50) + Pap q5y (55 - 60) | 4,384 | 0 | 1,859 | 2,524 | 25.88977 | 72.03 | 25.88165 | 24.54293 | 1.31140 | 0.02732 | Dom |
| 34 | VIA q5Y (30-45) + Pap q5y (50 - 65) | 4,507 | 0 | 1,975 | 2,532 | 25.88978 | 72.82 | 25.88165 | 24.54325 | 1.31106 | 0.02734 | Dom |
| 35 | Pap q5y (20 - 60) | 5,580 | 0 | 3,059 | 2,521 | 25.88979 | 73.84 | 25.88176 | 24.54244 | 1.31217 | 0.02716 | Dom |
| 36 | VIA q5Y (30-50) + Pap q5y (55 - 65) | 4,465 | 0 | 1,969 | 2,496 | 25.88986 | 72.82 | 25.88184 | 24.54458 | 1.31031 | 0.02696 | Dom |
| 37 | VIA q5Y (25-50) | 4,530 | 0 | 2,063 | 2,467 | 25.88985 | 71.24 | 25.88194 | 24.54281 | 1.31247 | 0.02666 | Dom |
| 38 | Pap q5y (20 - 65) | 5,661 | 0 | 3,169 | 2,492 | 25.88988 | 74.63 | 25.88196 | 24.54412 | 1.31106 | 0.02678 | Dom |
| 39 | VIA q5Y (30-55) + Pap q5y (60 - 65) | 4,435 | 0 | 1,963 | 2,472 | 25.88991 | 72.82 | 25.88197 | 24.54552 | 1.30976 | 0.02670 | Dom |
| 40 | VIA q5Y (20-50) | 5,042 | 0 | 2,674 | 2,369 | 25.89008 | 72.13 | 25.88252 | 24.54593 | 1.31108 | 0.02550 | Dom |
| 41 | VIA q5Y (25-55) | 4,576 | 0 | 2,231 | 2,345 | 25.89017 | 72.11 | 25.88268 | 24.54806 | 1.30933 | 0.02530 | Dom |
| 42 | VIA q5Y (25-45) + Pap q5y (50 - 60) | 4,736 | 0 | 2,382 | 2,354 | 25.89018 | 72.96 | 25.88268 | 24.54824 | 1.30910 | 0.02535 | Dom |
| 43 | VIA q5Y (25-45) + Pap q5y (50 - 65) | 4,817 | 0 | 2,491 | 2,325 | 25.89027 | 73.75 | 25.88288 | 24.54991 | 1.30800 | 0.02498 | Dom |
| 44 | VIA q5Y (25-50) + Pap q5y (55 - 65) | 4,774 | 0 | 2,485 | 2,290 | 25.89036 | 73.75 | 25.88307 | 24.55123 | 1.30725 | 0.02459 | Dom |
| 45 | VIA q5Y (25-55) + Pap q5y (60 - 65) | 4,745 | 0 | 2,479 | 2,266 | 25.89041 | 73.75 | 25.88320 | 24.55217 | 1.30670 | 0.02433 | Dom |
| 46 | VIA q5Y (20-55) | 5,088 | 0 | 2,841 | 2,247 | 25.89040 | 73.01 | 25.88326 | 24.55117 | 1.30794 | 0.02414 | Dom |
| 47 | VIA q5Y (20-45) + Pap q5y (50 - 60) | 5,248 | 0 | 2,992 | 2,256 | 25.89041 | 73.85 | 25.88326 | 24.55135 | 1.30771 | 0.02419 | Dom |
| 48 | VIA q5Y (20-50) + Pap q5y (55 - 60) | 5,206 | 0 | 2,986 | 2,220 | 25.89049 | 73.85 | 25.88345 | 24.55270 | 1.30695 | 0.02380 | Dom |
| 49 | VIA q5Y (20-50) + Pap q5y (55 - 60) | 5,206 | 0 | 2,986 | 2,220 | 25.89049 | 73.85 | 25.88345 | 24.55270 | 1.30695 | 0.02380 | Dom |
| 50 | VIA q5Y (20-45) + Pap q5y (50 - 65) | 5,329 | 0 | 3,102 | 2,227 | 25.89050 | 74.64 | 25.88346 | 24.55302 | 1.30661 | 0.02382 | Dom |
| 51 | Vac 13Y boost q10 | 5,240 | 3,145 | 0 | 2,095 | 25.89050 | 65.75 | 25.88351 | 25.17302 | 0.68767 | 0.02283 | Dom |
| 52 | Vac 13Y boost q15 | 4,762 | 2,668 | 0 | 2,095 | 25.89050 | 65.75 | 25.88351 | 25.17302 | 0.68767 | 0.02283 | Dom |
| 53 | Vac 13Y boost q20 | 4,531 | 2,436 | 0 | 2,095 | 25.89050 | 65.75 | 25.88351 | 25.17302 | 0.68767 | 0.02283 | Dom |
| 54 | Vac 13Y LT | 4,114 | 2,020 | 0 | 2,094 | 25.89050 | 65.75 | 25.88351 | 25.17302 | 0.68767 | 0.02283 | 2,407.37 |
| 55 | VIA q5Y (20-50) + Pap q5y (55 - 65) | 5,287 | 0 | 3,095 | 2,191 | 25.89058 | 74.64 | 25.88365 | 24.55435 | 1.30587 | 0.02343 | Dom |
| 56 | VIA q5Y (20-55) + Pap q5y (60 - 65) | 5,257 | 0 | 3,090 | 2,168 | 25.89064 | 74.64 | 25.88378 | 24.55529 | 1.30531 | 0.02317 | Dom |
| 57 | Vac 12Y boost q10 | 5,328 | 3,303 | 0 | 2,025 | 25.89066 | 65.75 | 25.88391 | 25.20500 | 0.65686 | 0.02205 | Dom |
| 58 | Vac 12Y boost q15 | 4,823 | 2,798 | 0 | 2,025 | 25.89066 | 65.75 | 25.88391 | 25.20500 | 0.65686 | 0.02205 | Dom |
| 59 | Vac 12Y boost q20 | 4,578 | 2,553 | 0 | 2,025 | 25.89066 | 65.75 | 25.88391 | 25.20500 | 0.65686 | 0.02205 | Dom |
| 60 | Vac 12Y LT | 4,139 | 2,114 | 0 | 2,025 | 25.89066 | 65.75 | 25.88391 | 25.20500 | 0.65686 | 0.02205 | Dom |
| 61 | Vac 11Y boost q10 | 5,400 | 3,447 | 0 | 1,953 | 25.89083 | 65.75 | 25.88433 | 25.23885 | 0.62424 | 0.02123 | Dom |
| 62 | Vac 11Y boost q15 | 4,863 | 2,910 | 0 | 1,953 | 25.89083 | 65.75 | 25.88433 | 25.23885 | 0.62424 | 0.02123 | Dom |
| 63 | Vac 11Y boost q20 | 4,604 | 2,651 | 0 | 1,953 | 25.89083 | 65.75 | 25.88433 | 25.23885 | 0.62424 | 0.02123 | Dom |
| 64 | Vac 11Y LT | 4,141 | 2,189 | 0 | 1,953 | 25.89083 | 65.75 | 25.88433 | 25.23885 | 0.62424 | 0.02123 | 4,658.65 |
| 65 | Vac 11Y LT + VIA q5Y (35-45) | 4,553 | 2,189 | 893 | 1,472 | 25.89196 | 68.58 | 25.88716 | 25.25597 | 0.61518 | 0.01600 | 33,126.39 |
| 66 | Vac 11Y LT + Pap q5y (35 - 55) | 4,928 | 2,189 | 1,305 | 1,434 | 25.89207 | 70.38 | 25.88743 | 25.25810 | 0.61376 | 0.01558 | Dom |
| 67 | Vac 11Y LT + Pap q5y (35 - 60) | 5,037 | 2,189 | 1,443 | 1,405 | 25.89216 | 71.23 | 25.88762 | 25.25952 | 0.61286 | 0.01523 | Dom |
| 68 | Vac 11Y LT + Pap q5y (35 - 65) | 5,131 | 2,189 | 1,552 | 1,390 | 25.89221 | 72.04 | 25.88772 | 25.26037 | 0.61231 | 0.01505 | Dom |
| 69 | Vac 11Y LT + VIA q5Y (35-50) | 4,660 | 2,189 | 1,095 | 1,377 | 25.89219 | 69.49 | 25.88772 | 25.25973 | 0.61303 | 0.01496 | 39,189.58 |
| 70 | Vac 11Y LT + VIA q5Y (30-45) | 4,879 | 2,189 | 1,315 | 1,376 | 25.89219 | 69.53 | 25.88773 | 25.25917 | 0.61363 | 0.01493 | Dom |
| 71 | Vac 11Y LT + Pap q5y (30 - 55) | 5,285 | 2,189 | 1,742 | 1,355 | 25.89226 | 71.33 | 25.88791 | 25.26075 | 0.61247 | 0.01469 | Dom |
| 72 | Vac 11Y LT + VIA q5Y (35-55) | 4,765 | 2,189 | 1,260 | 1,316 | 25.89235 | 70.38 | 25.88809 | 25.26234 | 0.61147 | 0.01428 | 45,212.57 |
| 73 | Vac 11Y LT + VIA q5Y (35-45) + Pap q5y (50 - 60) | 4,920 | 2,189 | 1,411 | 1,320 | 25.89236 | 71.24 | 25.88809 | 25.26243 | 0.61135 | 0.01430 | Dom |
| 74 | Vac 11Y LT + Pap q5y (30 - 60) | 5,394 | 2,189 | 1,880 | 1,326 | 25.89235 | 72.19 | 25.88810 | 25.26217 | 0.61158 | 0.01435 | Dom |
| 75 | Vac 11Y LT + VIA q5Y (35-50) + Pap q5y (55 - 60) | 4,895 | 2,189 | 1,404 | 1,302 | 25.89240 | 71.24 | 25.88819 | 25.26310 | 0.61098 | 0.01411 | Dom |
| 76 | Vac 11Y LT + VIA q5Y (35-45) + Pap q5y (50 - 65) | 5,015 | 2,189 | 1,520 | 1,306 | 25.89240 | 72.04 | 25.88819 | 25.26327 | 0.61080 | 0.01412 | Dom |
| 77 | Vac 11Y LT + Pap q5y (30 - 65) | 5,489 | 2,189 | 1,989 | 1,311 | 25.89240 | 72.99 | 25.88820 | 25.26301 | 0.61102 | 0.01417 | Dom |
| 78 | Vac 11Y LT + VIA q5Y (25-45) | 5,295 | 2,189 | 1,821 | 1,285 | 25.89241 | 70.49 | 25.88827 | 25.26208 | 0.61229 | 0.01389 | Dom |
| 79 | Vac 11Y LT + VIA q5Y (35-50) + Pap q5y (55 - 65) | 4,989 | 2,189 | 1,512 | 1,288 | 25.89244 | 72.04 | 25.88829 | 25.26392 | 0.61043 | 0.01393 | Dom |
| 80 | Vac 11Y LT + VIA q5Y (30-50) | 4,987 | 2,189 | 1,517 | 1,281 | 25.89242 | 70.45 | 25.88829 | 25.26291 | 0.61149 | 0.01389 | Dom |
| 81 | Vac 11Y LT + VIA q5Y (35-55) + Pap q5y (60 - 65) | 4,971 | 2,189 | 1,506 | 1,276 | 25.89247 | 72.04 | 25.88835 | 25.26439 | 0.61016 | 0.01380 | Dom |
| 82 | Vac 11Y LT + Pap q5y (25 - 55) | 5,734 | 2,189 | 2,265 | 1,280 | 25.89244 | 72.30 | 25.88836 | 25.26317 | 0.61135 | 0.01384 | Dom |
| 83 | Vac 11Y LT + VIA q5Y (20-45) | 5,855 | 2,189 | 2,422 | 1,244 | 25.89250 | 71.44 | 25.88851 | 25.26339 | 0.61171 | 0.01341 | Dom |
| 84 | Vac 11Y LT + Pap q5y (25 - 60) | 5,842 | 2,189 | 2,403 | 1,251 | 25.89253 | 73.15 | 25.88855 | 25.26459 | 0.61046 | 0.01350 | Dom |
| 85 | Vac 11Y LT + Pap q5y (20 - 55) | 6,322 | 2,189 | 2,887 | 1,246 | 25.89252 | 73.24 | 25.88856 | 25.26426 | 0.61086 | 0.01344 | Dom |
| 86 | Vac 11Y LT + Pap q5y (25 - 65) | 5,937 | 2,189 | 2,511 | 1,236 | 25.89258 | 73.95 | 25.88865 | 25.26543 | 0.60990 | 0.01331 | Dom |
| 87 | Vac 11Y LT + VIA q5Y (30-55) | 5,091 | 2,189 | 1,682 | 1,221 | 25.89258 | 71.34 | 25.88866 | 25.26551 | 0.60993 | 0.01321 | 64,609.76 |
| 88 | Vac 11Y LT + VIA q5Y (30-45) + Pap q5y (50 - 60) | 5,247 | 2,189 | 1,833 | 1,225 | 25.89259 | 72.19 | 25.88866 | 25.26561 | 0.60981 | 0.01324 | Dom |
| 89 | Vac 11Y LT + Pap q5y (20 - 60) | 6,431 | 2,189 | 3,025 | 1,217 | 25.89261 | 74.10 | 25.88875 | 25.26568 | 0.60997 | 0.01309 | Dom |
| 90 | Vac 11Y LT + VIA q5Y (30-50) + Pap q5y (55 - 60) | 5,222 | 2,189 | 1,826 | 1,207 | 25.89263 | 72.19 | 25.88876 | 25.26627 | 0.60944 | 0.01305 | Dom |
| 91 | Vac 11Y LT + VIA q5Y (30-45) + Pap q5y (50 - 65) | 5,341 | 2,189 | 1,942 | 1,211 | 25.89263 | 73.00 | 25.88876 | 25.26645 | 0.60926 | 0.01305 | Dom |
| 92 | Vac 11Y LT + VIA q5Y (25-50) | 5,403 | 2,189 | 2,023 | 1,191 | 25.89264 | 71.41 | 25.88883 | 25.26582 | 0.61015 | 0.01286 | Dom |
| 93 | Vac 11Y LT + Pap q5y (20 - 65) | 6,525 | 2,189 | 3,134 | 1,202 | 25.89265 | 74.90 | 25.88885 | 25.26652 | 0.60942 | 0.01291 | Dom |
| 94 | Vac 11Y LT + VIA q5Y (30-50) + Pap q5y (55 - 65) | 5,316 | 2,189 | 1,935 | 1,193 | 25.89267 | 73.00 | 25.88886 | 25.26710 | 0.60890 | 0.01286 | Dom |
| 95 | Vac 11Y LT + VIA q5Y (30-55) + Pap q5y (60 - 65) | 5,298 | 2,189 | 1,928 | 1,181 | 25.89270 | 73.00 | 25.88892 | 25.26757 | 0.60862 | 0.01273 | Dom |
| 96 | Vac 11Y LT + VIA q5Y (20-50) | 5,962 | 2,189 | 2,624 | 1,150 | 25.89274 | 72.36 | 25.88907 | 25.26713 | 0.60957 | 0.01237 | Dom |
| 97 | Vac 11Y LT + VIA q5Y (25-55) | 5,507 | 2,189 | 2,188 | 1,131 | 25.89280 | 72.30 | 25.88920 | 25.26842 | 0.60860 | 0.01218 | Dom |
| 98 | Vac 11Y LT + VIA q5Y (25-45) + Pap q5y (50 - 60) | 5,663 | 2,189 | 2,339 | 1,135 | 25.89280 | 73.15 | 25.88920 | 25.26852 | 0.60848 | 0.01220 | Dom |
| 99 | Vac 11Y LT + VIA q5Y (25-45) + Pap q5y (50 - 65) | 5,757 | 2,189 | 2,448 | 1,121 | 25.89285 | 73.96 | 25.88930 | 25.26935 | 0.60793 | 0.01202 | Dom |
| 100 | Vac 11Y LT + VIA q5Y (25-50) + Pap q5y (55 - 65) | 5,732 | 2,189 | 2,440 | 1,103 | 25.89289 | 73.96 | 25.88939 | 25.27001 | 0.60756 | 0.01183 | Dom |
| 101 | Vac 11Y LT + VIA q5Y (20-55) | 6,067 | 2,189 | 2,789 | 1,089 | 25.89289 | 73.25 | 25.88944 | 25.26973 | 0.60802 | 0.01169 | Dom |
| 102 | Vac 11Y LT + VIA q5Y (20-45) + Pap q5y (50 - 60) | 6,222 | 2,189 | 2,940 | 1,094 | 25.89290 | 74.10 | 25.88944 | 25.26983 | 0.60789 | 0.01172 | Dom |
| 103 | Vac 11Y LT + VIA q5Y (25-55) + Pap q5y (60 - 65) | 5,714 | 2,189 | 2,434 | 1,091 | 25.89291 | 73.96 | 25.88946 | 25.27047 | 0.60728 | 0.01170 | Dom |
| 104 | Vac 11Y LT + VIA q5Y (20-50) + Pap q5y (55 - 60) | 6,197 | 2,189 | 2,933 | 1,076 | 25.89294 | 74.10 | 25.88954 | 25.27049 | 0.60752 | 0.01153 | Dom |
| 105 | Vac 11Y LT + VIA q5Y (20-50) + Pap q5y (55 - 60) | 6,197 | 2,189 | 2,933 | 1,076 | 25.89294 | 74.10 | 25.88954 | 25.27049 | 0.60752 | 0.01153 | Dom |
| 106 | Vac 11Y LT + VIA q5Y (20-45) + Pap q5y (50 - 65) | 6,317 | 2,189 | 3,049 | 1,079 | 25.89294 | 74.91 | 25.88954 | 25.27066 | 0.60734 | 0.01153 | Dom |
| 107 | Vac 11Y LT + VIA q5Y (20-50) + Pap q5y (55 - 65) | 6,292 | 2,189 | 3,041 | 1,062 | 25.89298 | 74.91 | 25.88963 | 25.27131 | 0.60698 | 0.01134 | Dom |
| 108 | Vac 11Y LT + VIA q5Y (20-55) + Pap q5y (60 - 65) | 6,274 | 2,189 | 3,035 | 1,050 | 25.89301 | 74.91 | 25.88970 | 25.27178 | 0.60670 | 0.01121 | Dom |
